# Allelopathic Properties of Lamiaceae Species: Prospects and Challenges to Use in Agriculture

**DOI:** 10.3390/plants11111478

**Published:** 2022-05-31

**Authors:** A. K. M. Mominul Islam, Thiti Suttiyut, Md. Parvez Anwar, Abdul Shukor Juraimi, Hisashi Kato-Noguchi

**Affiliations:** 1 Department of Agronomy, Bangladesh Agricultural University, Mymensingh 2202, Bangladesh; parvezanwar@bau.edu.bd; 2 Department of Horticulture and Landscape Architecture, Purdue University, 625 Agriculture Mall Dr, West Lafayette, IN 47907, USA; tsuttiyu@purdue.edu; 3 Purdue Center of Plant Biology, Purdue University, West Lafayette, IN 47907, USA; 4 Department of Crop Science, Faculty of Agriculture, Universiti Putra Malaysia, Serdang 43400, Selangor, Malaysia; ashukur@upm.edu.my; 5 Department of Applied Biological Science, Faculty of Agriculture, Kagawa University, Miki 761-0795, Japan; kato.hisashi@kagawa-u.ac.jp

**Keywords:** allelochemicals, ecology, biodiversity, green agriculture, natural products, weed management

## Abstract

Herbicide resistance due to the increasing reliance on herbicides is a near-term challenge for the world’s agriculture. This has led to a desire to develop new herbicides with a novel mode of action, to address resistance in weed species. Lamiaceae, a large dicotyledonous plant family, is very well known for the multitudinous pharmacological and toxicological properties of its member species. Moreover, many species of this family are significant for their allelopathic activity in natural and laboratory settings. Thus, plants in Lamiaceae have the potential to be sources of alternative herbicides. However, gaps in our knowledge need to be addressed prior to adopting these allelopathic activities in agriculture. Therefore, we review the existing state of knowledge about the Lamiaceae family, the reported allelopathic properties of plant extracts, and their isolated allelochemicals under laboratory, greenhouse, and field conditions. In addition, we offer a perspective on existing challenges and future opportunities for adopting the allelopathic properties of Lamiaceae plant species for green agriculture.

## 1. Introduction

The direct or indirect detrimental or advantageous effects of a plant to its neighboring plants, or the plant itself, through the release of chemical substances are known as allelopathy, and the released substances are called allelochemicals [1]. Allelochemicals are present in nearly all plant tissues, including leaves, stems, rhizomes, roots, flowers, pollen, fruits, and seeds [2]. These allelochemicals are released into the environment through leaf or stem leaching (through precipitation), volatility (predominantly in semi-arid and arid conditions), root secretion, and tissue degradation by microorganisms [3]. Some allelochemicals can be released together and may exert toxicities in an additive or synergistic manner [4]. Upon release, allelochemicals may either inhibit or stimulate the growth of surrounding plants [2].

The present agricultural production system is impossible to imagine without the application of synthetic chemical pesticides. The high efficacy, cost-effectiveness, rapid return, flexibility, and easy accessibility of pesticides are the main reasons for such dependence [5]. The labor shortage due to outmigration from agriculture to other sectors also fuels the flames [6]. However, inappropriate dosages, application times and techniques, and/or storage practices [7] can cause various adverse effects for off-target organisms and farmers’ health [8,9]. Continued use of the same pesticides has also increased the rate at which pests develop resistance to the available chemical active ingredients. For example, to date, a total of 512 unique cases of herbicide-resistant weeds have been reported in 96 crops from 71 countries [10]. Going ahead, developing new pesticides will be restricted by harsh environmental, toxicological, and regulatory requirements [11]. This backdrop opens the door to producing natural product-based pesticides. In this context, the allelopathic properties of plants or their isolated allelochemicals may play a vital role. Although the inhibitory properties of the allelochemicals against test species are the main focus for most of the allelopathic studies [12], the biostimulatory activity of these chemicals to the target species at lower doses has also attracted the attention of many researchers [13].

Therefore, it is worth exploring the use of allelopathy in agriculture and studying the determinants preventing it from being implemented as a chemical tool. Lamiaceae is a large plant family and has been intensively studied and used in many applications [14,15]. Moreover, many species in Lamiaceae exhibit strong allelopathy. Hence, this review provides an overview of the present state of knowledge about the allelopathic properties of Lamiaceae plant species and their bioactive substances. This review also addresses the challenges and prospects of using Lamiaceae plants or their bioactive compounds in agriculture and the gaps for further research.

## 2. Lamiaceae in Brief

Lamiaceae (also known as Labiatae or the mint family) is a dicotyledonous aromatic plant family of the Angiosperm order Tubiflorae [16], comprising more than 250 genera and 7000 species [17]. The family is commonly distributed in temperate regions. It is one of the most commercially important plant families, and known for its pharmacological, pharmaceutical, and toxicological properties (Table 1). Most of the species are shrubby or herbaceous in nature, but a limited number of *Hyptis* and *Leucospectrum* are small trees; while *Gomphostemma* is a rain forest tree genus and a few species of *Scutellaria* are climbers [16]. Mainly they are xerophytes, but a few are hygrophytes, e.g., *Stachys palustris* [18,19]. The most distinct feature of Lamiaceae is being aromatic, because of the presence of significant quantities of volatile oils e.g., terpenes and their oxygenated derivatives, which are commercially important. Alkaloids are hardly present in this family. Some other substances such as glycosides, saponins, and resins are occasionally present [20]. The presence of these chemicals may confer economic value, and the toxic potential or allelopathic properties make this family one of the most interesting plant families to researchers [21]. A significant number of plant species of this family have medicinal value [20,21,22,23,24,25]. Plants belonging to the *Teucrium*, *Salvia*, *Dracocephalus*, *Thymus*, *Coleus*, *and Lavandula* genera are considered important ornamental species. Besides these, around 175 species of 45 genera of this family are considered weeds in different parts of the world [26].

A large number of Lamiaceae plant species, such as *Salvia officinalis* L. (Sage) [27], *Thymus vulgaris* L. (Thyme) [28], *Calamintha nepeta* L. (Lesser Calamint) [29], *Leucas aspera* Linn. (Thumbai) [30], *Origanum vulgare* L. (Oregano) [31], *Hyptis suaveolence* L. (Pignut) [32], *Satureja hortensis* L. (Summer Savory) [33,34], *Nepeta meyeri* Benth. (Catmint) [35], *Rosmarinus officinalis* L. (Rosemary) [36,37,38,39], and *Tectona grandis* L.f. (Teak) [40] show allelopathic and/or phytotoxic potential. The phytotoxic effect of their extracts, especially essential oils (EOs), was linked to the presence of volatile bioactive compounds [41,42,43].

## 3. Allelopathy of Major Lamiaceae Genera

### 3.1. Salvia (Sage)

*Salvia* is a well-known genus of Lamiaceae that consists of nearly 1000 species. The “*salvia* phenomenon” is one of the best-known examples of allelopathy (Table 2 and Table 3). Current allelopathic research gained momentum after 1964, when a picture of *Salvia leucophylla* inhibition zones appeared on the cover page of the journal Science [119]. Muller et al. [120] reported the volatile growth inhibitors of *Salvia leucophylla* and *S. apiana*. Two years later, Muller [121] isolated the allelopathic substances of *S. leucophylla* that are responsible for its growth-suppressive properties. After Muller, many researchers investigated the allelopathic activity and allelopathic substances of the genus *Salvia* [122,123]. The crude extract, essential oils (EOs), and isolated allelochemicals of *Salvia* spp. showed growth inhibitory/stimulatory activity for the target plant species. A summary of the allelopathic activity of *Salvia* spp. reported elsewhere is presented in Table 2 and Table 3. Although terpenoids are identified as the major allelopathic substances of *Salvia* spp., phenolic compounds and fatty acids (two common groups of allelochemicals) are also reported in *Salvia macrochylamis* extracts [124] (Table 3). Bisio et al. [125] identified 13 clerodane diterpenoids from *Salvia miniata* Fernald, whereas Martino et al. [126] identified 88 compounds from the essential oils of *Salvia africana* L., *Salvia greggii* A. Gray, *Salvia elegans* Vahl, *Salvia munzii* Epling, and *Salvia mellifera* Green. The amount of monoterpenoids and sesquiterpenoids are very similar in *S. africana*; while, in other species, the percentage of monoterpenoid is higher than the sesquiterpenoids. On the other hand, Nishida et al. [127] identified five volatile monoterpenoids: camphor, α-pinene, β-pinene, 1,8-cineole, and camphene from *Salvia leucophylla*, among them the volatile monoterpenoids camphor, 1,8-cineole are considered to be responsible for the “*Salvia* phenomenon” in natural settings [128].

*Salvia* extracts have insecticidal, antimicrobial, and antifungal activities. For example, a crude acetone extract of the aerial parts of *Salvia moorcraftiana* Wall. had moderate antifungal activity against animal and plant pathogens [129]. *Salvia sclarea* aqueous extract had a toxic effect on *Trialeurodes vaporariorum* (whitefly) with 57% mortality [130]. Zhang et al. [206] isolated and identified neo-przewaquinone A (a potent algicidal compound) from *Salvia miltiorrhiza* extract and reported this compound caused morphologic damage or lysis to cells, increased malondialdehyde content, and decreased the soluble protein content, total antioxidant, and superoxide dismutase activity; and significantly inhibited three photosynthesis-related genes (*psaB*, *psbD*, and *rbcL*) of *Microcystis aeruginosa*. On the other hand, Fraga et al. [207] identified demethylsalvicanol, 14-deoxycoleon U, demethylcryptojaponol, and a few other compounds from the roots of *Salvia broussonetii*. Among them, demethylsalvicanol and 14-deoxycoleon U were moderate and strong antifeedants to *Leptinotarsa decemlineata*, respectively, while demethylcryptojaponol was toxic to this insect.

### 3.2. Nepeta

The plant species of the genus *Nepeta* are generally known as “Catnip” or “Catmint”. The genus comprises more than 250 species. Although the species of this genus are usually distributed in North America, Europe, Asia, and Africa, the greatest species diversity is found in Asia (111 species) [53]. The extracts and essential oils of various *Nepeta* species have a wide spectrum of biological activities (Table 1). Mutlu et al. [209] published an image of the inhibition zone of *Nepeta meyeri* that did not allow the germination of other wild plant species in natural settings. Several researchers around the globe also reported the allelopathic activity of *Nepeta* spp. and identified the allelopathic substances responsible for their phytotoxic activity (Table 2 and Table 3).

The EOs from the aerial parts of *Nepeta cataria* L. showed phytotoxic activity to three noxious weeds (*Hordeum spontaneum* Koch, *Avena fatua* L., and *Taraxacum officinale*) and tree crops (*Lipidium sativum*, *Ocimum basilicum*, and *Nepeta cataria*) species, at different inhibition levels [247]. For example, with a concentration of 1200 μL L^−1^, 100% inhibition of seed germinations (complete inhibition) of all test species except in *H. spontaneum* were observed. At this concentration, 26% seed germination of *H. spontaneum* was observed. In addition, the germination percentages of A. *fatua* and *T. officinale* at 600 μL L^−1^ and *L. sativum* and *O. basilicum* at 1200 μL L^−1^ were completely inhibited (100%) [247]. Eom et al. [208] reported that the volatiles of *N*. × *faassenii* fresh foliage and its aqueous and methanol extracts significantly inhibited the seedling growth of curly cress (*Lepidium sativum*). They observed that *N*. × *faassenii* volatiles inhibited shoot and root growth by 48% and 44%, respectively, at 10 g of foliage, whereas the root and shoot growth of *L. sativum* was completely inhibited with the 20 g foliage treatment. On the other hand, aqueous extracts of *N*. × *faassenii* showed higher inhibition compared to methanolic extracts when similar dosages of extracts were applied. Complete (100%) inhibition on the germination of *L. sativum* was observed when exposed to concentrations of 0.5 mg mL^−1^ or more aqueous extracts and 1.0 mg mL^−1^ methanolic extracts, respectively. Furthermore, the aqueous foliage extracts exhibited greater activity in seedling growth reduction on a per-weight basis than methanolic extracts. Three phytotoxic compounds responsible for *N*. × *faassenii* phytotoxic activity: 2-(2-ethoxyethoxy)ethanol, alloaromadendrene, and *x*-cadinene, were isolated from the volatile mixture, methanolic extract, and the aqueous foliar extract [208].

Mutlu and Atici [135] observed that the roots and leaves extracts of *Nepeta meyeri* Benth. showed an allelopathic effect on the germination and seedling growth of barley and sunflower. On the other hand, both extracts significantly increased the seedling growth of canola, wheat, and safflower, especially at the lower concentrations (up to 0.5% of the extract). Interestingly, a neutral effect was found at higher concentrations only in wheat and being phytotoxic to other species. Their findings reported that the allelopathic activity of *N. meyeri* depends on the source of the plant extract (leaf or root), and the highest inhibitory activity was observed with leaf extracts. Mutlu et al. [209] identified a strong oxygenated monoterpene; 4aα,7α,7aβ-nepetalactone from the EOs of *Nepeta meyeri* Benth. aerial parts, as a major compound responsible for their phytotoxicity. This monoterpene showed concentration-dependent inhibitory activity on the germination and growth of weed species, including *Amaranthus retroflexus* L., *Bromus intermedius* Guss., *Bromus danthoniae* Trin., *Cynodon dactylon* L., *Chenopodium album* L., and *Lactuca serriola* L. Kordali et al. [35], on the other hand, evaluated the phytotoxic activities of the essential oil, n-hexane, chloroform, acetone, and methanol extracts of the aerial parts and roots of *N. meyeri* Benth. against the germination and growth of four weed species: *A. retroflexus*, *C. album*, *Cirsium arvense* L., and *Sinapsis arvensis* L. The essential oil of *N. meyeri* completely inhibited the germination of all weed seeds, although the extracts showed a wide variation in their inhibition of the seedling growth of the weed species. The extracts and EOs also showed phytotoxicity against these weeds. The acetone extract was the most effective compared to the other extracts. The mortality percentage ranged from 22–66% (48 h after application). Finally, Kordali et al. [35] isolated the main compound from the EOs and n-hexane extract, and reported 4aα,7α,7aβ-nepetalactone (>80%) as the main allelopathic compound responsible for the phytotoxic activity of *N. meyeri*. The genotoxical activity of 4aα,7α,7aβ-nepetalactone isolated from the EOs of the aerial parts of *N. meyeri* was also reported against the weeds, *Bromus danthoniae* and *Lactuca serriola*; and crops, *Brassica napus* and *Zea mays* [212].

The EOs of *Nepeta curviflora* Boiss. contained β-caryophyllene, caryophyllene oxide, (*E*)-β-farnesene, and (*Z*)-β-farnesene; *Nepeta nuda* L. subsp. albiflora (Boiss.) Gams. contained β-bisabolene, pulegone, (*E*,*Z*)-nepetalactone, (*E*)-β-farnesene, and caryophyllene oxide, while *Nepeta nuda* L. subsp. albiflora (collected from different areas) contained hexadecanoic acid, β-bisabolene, caryophyllene oxide, pulegone, and (*E*,*Z*) nepetalactone. All these EOs showed phytotoxic activity on the germination and initial root growth of *Lepidium sativum* L. and *Raphanus sativus* L. [211]. Formisano et al. [248] isolated 75 compounds, which comprised 96.8% of the total EOs; where spathulenol (15.1%), hexadecanoic acid (14%), δ-cadinene (5.5%), and α-copaene (4.5%) were the main compounds. In addition, the oil was constituted mainly by sesquiterpenes (45.9%), among which 27.6% was sesquiterpene hydrocarbons and 18.3% was oxygenated sesquiterpenes. Bozok [213], on the other hand, isolated 41 compounds from the EOs of the aerial parts of *Nepeta flavida*, where linalool (37.6%) and 1,8-cineole (30.8%) were the most abundant compounds that completely inhibited the germination of *Lepidium sativum*, *Raphanus sativus*, and *Eruca sativa* at concentrations of 4.0 and 8.0 μL ml^−1^. The antimicrobial activity of the chloroform fraction of *Nepeta juncea* against *Salmonella typhi* was also reported by Hussain et al. [249].

### 3.3. Ocimum

The genus *Ocimum*, commonly known as basil, comprises around 60 species. They are widely distributed throughout tropical and subtropical Asia [137,141,250]. Due to its numerous pharmacological properties (Table 1), the plant is designated as “Holy Basil” in India. It is grown in the front of houses and temples by the Hindus, besides being cultivated for EO production. The EOs of *Ocimum* spp. have either phenolic constituents, eugenol, thymol, or sesquiterpene alcohols as single major oil constituents, or terpene compounds as minor constituents [55,56].

Culpeper [251] reported that *Ocimum* and *Ruta graveolens* never grow together, nor near one another; this was probably the first report about the allelopathic activity of any plant species of the Lamiaceae family. Since that, several reports have been found in the literature about the allelopathic activity of *Ocimum* species (Table 2 and Table 3). Samunder and Megh [252] reported the autotoxic activity of *Ocimum* leachate (collected from soil). Likewise, soil incorporation of *Ocimum* root/shoot biomass in a 1:12 ratio (*Ocimum*: soil) had no inhibitory effect on the emergence of *Desmodium tortuosum*, *Sorghum halepense*, *Morrenia odorata*, *Amaranthus retroflexus*, *Senn*a *obtusifolia*, *Bidens pilosa*, and *Cyperus esculentus*. However, *Ocimum* suppressed the growth of all those weed species, except *Cyperus esculentus*. The aqueous extract of *Ocimum basilicum* L. aerial parts showed a dose-dependent inhibition on the germination of *Sorghum bicolor* L. Moench, *Pennisetum glaucum* L. R. Br., *Zea mays* L., and *Triticum vulgare* L. [138]. A similar type of inhibition was also observed on the plant height, root length, seedling fresh, and dry weight of those crop seedlings when powder of its aerial parts was used.

Mota et al. [253] evaluated the insecticidal properties of EO of *Ocimum micranthum* Willd against *Aleurodicus cocois* (whitefly). They observed that the EO at 1% concentration was highly toxic to *A. cocois*, with faster mortality (6.82 h). They also identified eugenol, elemicin, and beta-caryophyllene as the major allelochemicals that were responsible for the insecticidal properties of *O. micranthum* essential oil.

### 3.4. Lavandula (Lavender)

The genus *Lavandula* comprises 40 species and around 80 wild infra-specific taxa and hybrids [254,255]. The *Lavandula* spp. is one of the important aromatic plants cultivated near the Mediterranean basin. *Lavandula angustifolia* Mill., *Lavandula stoechas* L., *Lavandula latifolia* Medik., and *Lavandula* × *intermedia* are the four major species of lavender. The EOs of *Lavandula* species are very well known for their myriad of pharmacological, toxicological, and pharmaceutical properties [146,147,217,218,256,257] (Table 1).

Lavender extracts and their EOs have allelopathic properties, as reported by many researchers worldwide (Table 2, Table 3 and Table 4). The aqueous extracts of *L. officinalis* shoot were allelopathic to weed species, including *Amaranthus retroflexus* and *Chenopodium murale* germination and growth [258]. Likewise, aqueous extracts of *L. stoechas* were phytotoxic to the germination and growth of both crop (*Triticum aestivum*) and weed (*Phalaris minor*) species [259]. The EOs of *L. stoechas* and *L. angustifolia* were also reported as phytotoxic to the seedling growth of *A. retroflexus* and *Portulaca oleracea*, two common weeds of *Lycopersicon esculentum* and *Gossypium* spp. [260]. Uremis et al. [261] reported that the volatile compounds of *L. angustifolia* negatively affect the germination of the most common weed species: *Xanthium strumarium* L., *Avena sterilis* L., and *Phalaris brachystachys* L.

Haig et al. [146] examined the allelopathic activity of *Lavandula* spp. against *Lolium rigidum*. They reported that the extract at the concentration of 10% completely inhibited the root growth of the test species and that coumarin was the main allelochemical responsible for its phytotoxicity. The EOs (linalool and linalyl acetate) of *Lavandula* × *hybrida* Rev. were evaluated against the germination and seedling growth of two crops (*Triticum aestivum* L. and, *Hordeum vulgare* L.) and two weeds (*Lolium rigidum* L. and *Phalaris brachystachys* L.), where the EOs showed no effect on crop species but inhibited the germination and seedling growth of both weeds [218]. Similarly, *Lavandula angustifolia* Mill. EOs, terpinen-4-ol, lavandulol and linalyl acetate, and lavandulyl acetate completely inhibited the germination and root length of *Setaria verticillata* (L.) P. Beauv. at concentrations of 80, 160, and 320 nL mL^−1^, respectively [217]. Recently, two new tricyclic sesquiterpenoids, stoechanones A and B have been isolated from the organic extract of *Lavandula stoechas*, which exhibited strong phytotoxicity against the germination and seedling growth of *Amaranthus retroflexus* [219].

### 3.5. Mentha

The genus *Mentha* (commonly known as mint) has 61 species and hundreds of subspecies, varieties, and cultivars, which are widely distributed throughout the world, except South America and Antarctica [220,271,272]. The species of this genus are also well known for their relatively high content of EOs and a significant number of medicinal and pharmaceutical properties [271,272,273,274] (Table 1). The allelopathic properties of *Mentha* spp. from their different parts (leaves, stem, flowers, seeds, and roots) and fractions (essential oils, volatiles, below ground soils, dry biomass) under several experimental settings (laboratory, greenhouse, pot or field conditions) have been well documented, as shown in Table 2, Table 3 and Table 4. The major allelochemicals isolated from the EOs of *Mentha* spp. known to date are piperitone oxide and piperitenone oxide from *M. longifolia* (L.) Huds.; carvone, piperetenone oxide from *M. spicata* L.; (-)-menthol, menthone from *M*. × *piperita* L.; menthol, mentone, menthofuran from *M*. × *piperita* L. cv. Mitcham; pulegone, menthone from *M. pulegium* L. and *M*. × *piperita* and menthone/isomenthone from *M. spicata*, *M. crispa*, and *M. longifolia* (Table 2). In addition, *trans*-ferulic acid, hesperidin, ellagic acid, and sinapic acid have been isolated from the aqueous extract of *M*. × *piperita* L. cv. Mitcham [148,225]. All these compounds either inhibited (at high concentration) or stimulated (at low concentration) the germination, growth, and other morpho-physiological processes of different test species (Table 2). Maffei et al. [275] reported that an increase of *Mentha piperita* L. EOs concentration from 5 to 50 ppm decreased the membrane potential hyperpolarization of 10^–3^ mV, while concentrations from 100 to 900 ppm decreased the depolarization of membrane potential (from 5 to 110 mV). In another study, Skrzypek et al. [264] reported that increasing concentrations of aqueous extracts of *M*. × *piperita* L. decreased the chlorophyll *a*, while increasing the chlorophyll *b* content of *Helianthus annuus* L. Sarheed et al. [224] found that menthone disrupted the microtubules and induced mortality, linked with rapid permeabilization of the plasma membrane of both tobacco BY-2 cells and *Arabidopsis thaliana* seedlings.

In a pot experiment, direct incorporation of *M. spicata* L. plant residue into the soil could boost tomato tolerance against soil-borne fungi and soil fertility, and accordingly increased the yield and quality of the tomato [264]. Similarly, mixed cultivation of two *M*. × *piperita* varieties in glasshouse stimulated the leaf development of *Brassica oleracea* [265], and application of dry leaves of *M*. spp. in pots also stimulated the seedling emergence of *Brassica napus* [267]. By contrast, the introduction of *M. spicata* L. and *M. piperita* L. in crop rotation inhibited the plant height, biomass, photosynthetic rate, stomatal conductance, and relative chlorophyll content of maize [266].

### 3.6. Thyme

The genus *Thymus* comprises around 400 species, with several subspecies, varieties, sub-varieties, and forms. *Thymus* species are extensively used as a culinary herb in both fresh and dried forms [155,276]. The plants of this genus are widely used in different industries, due to their numerous pharmacological, pharmaceutical, cosmetic, perfume, and toxicological properties [276,277,278,279,280,281] (Table 1). *Thymus* species are also used for flavoring and preservation of different foods [279]. A significant number of articles are available in the literature explaining the allelopathic properties of *Thymus*.

Ali et al. [155] reported the inhibitory activity of *Thymus numidicus* Poir. root extract on the seedling growth of *Medicago sativa* and *Triticum aestivum*. The essential oils of *T. daenensis* strongly inhibited the germination percentage (IC_50_ 2.9 ppm) of *Lactuca sativa* and were lethal to the embryo (LC_50_ 7.2 ppm), while *T. transcaspicus* delayed the germination time of the same [3]. On the other hand, Ali et al. [227] reported the phytotoxic potential of *T. algeriensis* Boiss. Et Reut. leaf oils on the seedling growth of *M. sativa* and *Triticum aestivum*. They also reported that α-pinene (19.5%), 1,8-cineole (11.6%), and camphor (10.4%) were the major constituents of *T. algeriensis*. These oils were found to possess strong insecticidal activity (LC_50_ = 44.3–112.8 µL L^−1^ air) against the third instar larvae of cotton leafworm (*Spodoptera littoralis* Boisd.; Lepidoptera: Noctuidae). The EOs (mainly thymol, 60.48%) of *T. kotschyanus* showed phytotoxic activity (>500 ppm concentration) against *Amaranthus retroflexus* L. and *Panicum miliaceum* L., fungicidal activity (>500 ppm concentration) against *Aspergillus niger*, *Botrytis cinerea*, and *Penicillium expansum*, and insecticidal activity against *Oryzaephilus surinamensis* (LC_50_ = 4.78 µL L^−1^ air) [281]. The EOs of *T. vulgaris* (mainly thymol and carvacrol) showed insecticidal activity against *Alphitobius diaperinus* Panzer (Coleoptera, Tenebrionidae) at the early stage of growth [282].

### 3.7. Hyptis

The genus *Hyptis* is composed of 400 species that mainly occur in tropical America, but are also found in other regions of the world [283]. The species of this genus is traditionally used to treat various ailments [76,284] (Table 1). A few are considered as invasive weeds in some parts of the world, and possess allelopathic properties. For example, *Hyptis suaveolens* (L.) Poit is an important invader of tropical and sub-tropical regions, and that restricted the growth and establishment of other plant species near their clumps [77,285,286,287]. A plausible reason could be their allelopathic properties [78,160,164,288,289]. This statement is supported by the isolation and identification of a putative allelochemical 14α-hydroxy-13β-abiet-8-en-18-oic acid (suaveolic acid) from this plant species by Islam et al. [32].

A number species from this genus also have insecticidal and repellent properties. For example, Tripathi and Upadhyay [290] reported the insecticidal (LD_50_ value of 57.0 mg mg^−1^ weight of insect and 4.7 mg L^−1^ air) and repellent (% repellence ranged from 20.0 to 94.7% at 18.3 mg cm^−2^ for 5 h) properties of *H. suaveolens* leaf EOs on stored grain coleopteran pests, e.g., *Callosobruchus maculatus*, *Rhyzopertha dominica*, *Sitophilus oryzae*, and *Tribolium castaneum*. The major EOs responsible for this activity identified were sabinene (41.0%), terpinen-4-ol (12.31%), β-pinene (10.0%), and β-caryophyllene (8.0%). Conti et al. [291], on the other hand, reported the larvicidal (more than 90% mortality at concentration > 400 ppm) and repellent properties of *H. suaveolens* EOs against *Aedes albopictus* Skuse. Terpinolene was the major compound responsible for this activity. The insecticidal and repellent properties of *H. suaveolens* EOs against *Tenebroides mauritanicus* (L.) (peanut pest) were also reported by Adjou et al. [292].

The insecticidal properties of *H. Spicigera* Lam. were reported by Fragoso-Serrano et al. [293] and Noudjou et al. [294]. The Labdane type diterpenes 15,19-diacetoxy-2*R*,7*R*-dihydroxylabda-8(17), (13*Z*)-diene isolated from *H. spicigera* significantly inhibited the larval growth of the European corn borer [293]. Araújo et al. [283] reported the insecticidal properties of *H. martiusii* Benth EOs against *Aedes aegypti* larvae (vector of dengue fever) and *Bemisia argentifolii* (vector of white fly fruit plague). Whereas, the antifungal activity of *H. ovalifolia* leaves EOs was reported by Souza et al. [284].

### 3.8. Leucas

The genus *Leucas* comprises about 80 species, of which 43 species are available in India [295], whereas the highest species diversity is found in East Africa [79,296]. Similar to other genus of Lamiaceae, the species of *Leucas* have also been widely used by traditional healers to cure many human ailments [23,25], because of its many pharmaceuticals and toxicological properties [297] (Table 1). These genus features indicated its immense potential for the discovery of novel allelopathic compounds. Although the phytochemical study of *Leucas* started many years back, very few allelopathic studies with this plant have been done so far. Islam and Kato-Noguchi [169] reported the phytotoxic properties of *Leucas aspera* (Willd.) Link against several weeds. The methanol extract of *L. aspera* (at 100 mg DW equivalent extract mL^−1^) showed stronger phytotoxic activity against *Ehiconchloa crus-galli* [298]. Islam et al. [30] isolated and characterized an equilibrium (or inseparable) 3:2 mixture of two labdane type diterpenes, (rel 5*S*,6*R*,8*R*,9*R*,10*S*,13*S*,15*S*,16*R*)-6-acetoxy-9,13;15,16-diepoxy-15-hydroxy-16-methoxylabdane and (rel 5*S*,6*R*,8*R*,9*R*,10*S*,13*S*,15*R*,16*R*)-6-acetoxy-9,13;15,16-diepoxy-15-hydroxy-16-methoxylabdane from the aqueous methanol extract of *L. aspera*. This mixture inhibited the germination and seedling growth of allelopathic sensitive *Lepidum sativum* (IC_50_, 31 µM) and the most noxious weed, *Echinochloa crus-galli* (IC_50_, 80 µM), at concentrations higher than 30 and 3 µM, respectively.

### 3.9. Leonurus

Similar to the other genus of Lamiaceae, *Leonurus* species also has myriad medicinal properties (Table 1) and are used by herbalists to treat gynecological and obstetrical disorders in China, and anxiety and heart diseases in European countries [299]. To date, 24 species of *Leonurus* have been identified. *Leonurus japonicus* (also known as *L. heterophyllus*) and *L. cardiaca* are the distinctive species of Eastern Asia and Europe, respectively [299]. Some other dominant species of *Leonurus* include *L. japonicus*, *L. cardiaca*, *L. persicus*, *L. sibiricus*, *L. macranthus*, *L. turkestanicus*, and *L. glaucescens*.

Very little is known about the allelopathic and phytotoxic properties of the species from this genus. The few articles related to their allelopathy are mostly with *L. sibiricus*. In addition, most of the reports of *L. sibiricus* allelopathy have mainly been based on simple laboratory bioassay experiments (Table 2 and Table 3). Mandal [235] reported that caffeic acid, isolated from the root exudates, is responsible for the growth inhibitory activity of *L. sibiricus*. Almeida et al. [236] isolated three major allelopathic compounds, 3′-OH-genkwanin, rutin, and isoquercitrin, from the methanol extract of *L. sibiricus*. Other than these, Wu et al. [300] isolated (−)-loliolide from *L. japonicus*. The phytotoxic properties of loliolide (isolated from the species of other plant families) for different test species has been reported by a few researchers from Japan and China [301,302,303,304]. Moreover, Labdane diterpenoids are the typical compounds of *Leonurus* [299]. The mixture of the two members of this group is responsible for the allelopathic activity *Leucas aspera* [30]. A Labdane-type diterpene isolated from *Hyptis spicigera* inhibited the larval growth of the European corn borer [293], as reported earlier.

### 3.10. Origanum

*Origanum*, commonly known as oregano, is an important culinary genus of Lamiaceae. It comprises around 900 species and is distributed throughout the world [86,87]. Considering pharmacological, pharmaceutical, and toxicological properties, the species from this genus are not different from other Lamiaceae genus (Table 1). A number of research works have been conducted to explore the allelopathic potential of *Origanum* plants or their essential oils (Table 2 and Table 3).

Among the allelopathic substances identified to date from *Origanum* EOs, carvacrol, thymol, γ-terpinene, p-cymene, β-cymene, methyleugenol, myristicin, caryophyllene oxide, β-caryophyllene, and α-cadinol are the major compounds that showed inhibitory activity on the germination, growth, and physiological parameters of different test species at different inhibition values (Table 3). *Origanum syriacum*, *O. vulgare* ssp. vulgare L., *O. vulgare* ssp. hirtum, *O. acutidens*, *O. onites*, *O. compactum* Benth., and *O. majorana* L. are the dominant species of *Origanum* that showed allelopathic properties [3,37,174,175,176,177,178,179].

### 3.11. Rosmarinus (Rosemary)

*Rosmarinus*, another popular genus of Lamiaceae family, comprises three different species, e.g., *R. officinalis*, *R. eryocalix*, and *R. tomentosus*, mainly found in the western Mediterranean region. Similar to other genus of this family, *Rosmarinus* also has a number of medicinal properties (Table 1). However, the genus is very popular as a culinary herb and is used as a food flavoring or food preservative [89]. Few allelopathic reports of *Rosmarinus officinalis* were found in literature (Table 2). The major allelochemicals of *R. officinalis* EOs are α-pinene, 1,8-cineole, and piperitone, which significantly inhibited the germination and seedling growth of weed species, e.g., *Eleusine indica* (L.) *Gaertn*., *Cynodon dactylon* (L.) *Pers*., *digitaria sanguinalis* (L.) *Scop*., *Amaranthus retroflexus* L., and *Lolium perenne* [39,89]. Najem et al. [180] also reported the anti-cyanobacterial activity of *R. officinalis* essential oil on *Microcystis aeruginosa* and *Chroococcus minor*, where *M. aeruginosa* was more sensitive than *C. minor*. However, no reports related to the allelopathic properties of *R. officinalis* under field conditions were found in literature.

### 3.12. Hyssopus (Hyssop)

*Hyssopus* is a small aromatic medicinal genus of the Lamiaceae family, comprising only 70 species, of which *Hyssopus officinalis* L. is the most dominant [305]. Very little work has been conducted to explore the allelopathic activities of this genus so far. Dragoeva et al. [173] reported its allelopathic properties from a preliminary laboratory bioassay of *Hyssopus officinalis* on *Cucumis sativus* L., *Triticum aestivum* L., and *Allium cepa* L. However, Ortiz de Elguea-Culebras et al. [306] reported that 1,8-cineole (53%) and β-pinene (16%) are the major bio-active compounds of the EOs of *H. officinalis* that are insecticidal to *Spodoptera littoralis* (cotton leafworm). They also reported that *H. officinalis* EOs have no inhibitory effect on the germination of *Lactuca sativa* L. var. *Carrascoy* and *Lolium perenne* L., but have a slight inhibitory effect on the root and leaf growth of *L. perenne*.

### 3.13. Orthosiphon

*Orthosiphon* is another small Lamiaceae genus that comprises around 40 species, distributed throughout the tropical and sub-tropical Asia, Southern Africa, Madagascar, and some parts of Australia [100]. Some species of this genus have medicinal properties (Table 1); among them, *Orthosiphon aristatus*, *Orthosiphon thymiflorus*, *Orthosiphon pallidus*, and *Orthosiphon stamineus* are very well known [100]. To date, the allelopathic properties of only two species *Orthosiphon stamineus* and *Orthosiphon aristatus* have been reported in the literature. Suwitchayanon et al. [307] observed 75% root and 45% shoot growth inhibition of *Lactuca sativa* by the dried powder of *Orthosiphon aristatus* with a modified sandwich method, under laboratory conditions. While, the aqueous methanol extract of *Orthosiphon stamineus* showed inhibitory activity on the seedling growth of *Lepidium sativum* and *L. sativa*, and a novel allelopathic substance 13-epi-orthosiphol *N* was identified by Kato-Noguchi et al. [200]. This compound inhibited the root and shoot growth of *L. sativum* and *L. sativa* at concentrations higher than 10 µmol L^−1^, and the concentrations required for 50% growth inhibition ranged 41–102 µmol L^−1^ [200].

### 3.14. Tectona

*Tectona* is a genus of tropical hardwood trees and comprises three species: *T. grandis*, *T. hamiltoniana*, and *T. philippinensis* [104]. *T. hamiltoniana* and *T. philippinensis* are now considered endangered species and confined to Burma and the Philippines, respectively. The most common species, *T. grandis* is native to South and Southeast Asia and is widely distributed to tropical Asia, Africa, and Central and South America, due to its quality timber [308,309,310,311,312]. Besides its high-quality timber properties, the genus *Tectona* is well known for its ethnobotanical and toxicological properties [40,104].

Recently, Kato-Noguchi [104] reviewed the allelopathic properties of *Tectona grandis* L.f. The leachate, leaves, EOs, and underground soil of *T. grandis* had allelopathic properties and inhibited the morpho-physiological growth of several crop species (Table 2, Table 3 and Table 4). Kole et al. [313] applied the powder of fallen *T. grandis* leaves (100 g 7.2 m^−2^) in a wheat field and observed a 45% reduction in weed population at 21 days after application, but interestingly the powder did not affect the wheat growth. Macías et al. [245] reported two diterpenes; 2-oxokovalenic acid and 19-hydroxyferruginol from the water extract of the dried leaves of *Tectona grandis*, which inhibited the germination and growth of *Lactuca sativa*. The highest inhibitory effects on the germination were caused by 2-oxokovalenic acid, which showed activity values similar to the herbicide Logran^®^ at the highest concentrations (89% inhibition at 10^−3^ M concentration). However, both compounds stimulated the root growth of *Lycopersicum esculentum*, with values higher than 20% for all concentrations.

Lacret et al. [314] isolated naphthotectone from the dried leaves of *T. grandis* that inhibited the germination and seedling growth of *Triticum aestivum* L., *Allium cepa* L., *Lycopersicon esculentum* L., and *Lactuca sativa* L. Macías et al. [315] on the other hand isolated 3β-hydroxy-7,8-dihydro-β-ionol and 3β-hydroxy-7,8-dihydro-β-ionone from the dried leaves. Both compounds inhibited the seedling growth of *Triticum aestivum*, *Allium cepa*, and *Lycopersicum esculentum*. All these compounds showed an activity similar to the commercial herbicide Logran^®^. It is important to note that all the above mentioned bioactive compounds were isolated from the water extract of *T. grandis* dried leaves, and their activity was examined only against crop species. Hence, it may be necessary to evaluate the phytotoxic potential of those compounds against weed species.

### 3.15. Satureja (Savory)

*Satureja*, a well-known genus from the Mediterranean area, Asia and some parts of USA, includes more than 200 aromatic species [105]. The species of this genus has also traditionally been used for gastrointestinal cramps, diarrhea, nausea, muscle pains, and some other infectious diseases, because of their various medicinal properties [316] (Table 1). Due to their characteristic smell, a few species of this genus are also used for culinary purposes and herbal tea [317]. A negligible number of allelopathic reports of *Satureja* species have been published so far. The aqueous extract of *Satureja montana* L. [185,187], *Satureja thymbra* L. [186], *Satureja khuzestanica* Jamzad, *S. bachtiarica* Bunge, and *S. rechingeri* Jamzad [189], *Satureja hortensison* L. [190] aerial parts significantly inhibited the germination and growth of the tested species (Table 2). Beside these, two allelopathic compounds; carvacrol and γ-terpinene have been isolated from the aerial parts of *Satureja hortensis* L., which inhibited the germination and growth of *Lollium rigidum* L. and *Phalaris brachystachys* L. [218], and from its EOs, which inhibited the germination, root and shoot growth, and chlorophyll content of *Amaranthus retroflexus* and *Chenopodium album* [244]. In addition, Taban et al. [107] isolated carvacrol and thymol from the EOs of *Satureja* spp., *S. khuzestanica*, *S. bachtiarica*, *S. rechingeri*, and *S. spicigera*, which inhibited the germination and growth of *Secale cereale* and *Lycopersicon esculentum* at different inhibition values. Among them, EOs isolated from *S. khuzestanica* were highly phytotoxic and were suggested for bio-herbicide development [107].

Askun et al. [318] reported strong fungicidal effects of *S. icarica*, *S. coerulea*, and *S. cilicica* methanolic extracts at high concentrations of 6.3 to 12.5 mg mL^−1^ and fungistatic effects at lower concentrations. They identified carvacrol, hesperidin, and apigenin from *S. icarica*; rosmarinic acid, carvacrol, and caffeic acid from *S. coerulea*; and rosmarinic acid, hesperidin, and quercetin from *S. cilicica* methanolic extracts as the major compounds.

### 3.16. Conradina

*Conradina*, a small aromatic genus of the Lamiaceae family, is mainly found in the xeric environments with well drained sandy soil of the United States [109]. The family consists of six endemic species: Conradina *canescens* A. Gray, *C. cygniflora* C.E. Edwards, Judd, Ionta & Herring, *C. etonia* Kral & McCartney, *C. glabra* Shinners, *C. grandiflora* Small, and *C. verticillata* Jennison [319]. The species of this genus has colonizing ability in xeric disturbed soils [109]. Among the six species of *Conradina*, only the allelopathic potential of *C. canescens* has been reported in the literature to date.

Water leachate of *C. canescens* fresh leaves have germination and growth inhibitory potential, and eight monoterpenes: 1,8-cineole, camphor, borneol, myrtenal, myrtenol, α-terpineol, carveol, and carvone were identified from this leachate [320]. The saturated aqueous extract of these monoterpenes showed strong phytotoxicity against *Leptochloa dubia*, *Schizachyrium scoparium*, and *Lactuca sativa*, except 1,8-cineole, which did not affect *L. dubia* [321,322]. Dosoky [323] observed the inhibitory activity of *C. canescens* essential oil and isolated ursolic acid on the germination of *Lactuca sativa* and *Lolium perenne*. The presence of ursolic acid has a major role in the allelopathic potential of this species. This compound is thought to act as a natural detergent, by leading water-insoluble monoterpenes to form micelles, rendering them water-soluble; thus, boosting their ability to leach into rainwater for delivery into the soil [324]. Moreover, this compound helps co-solubilize the allelopathic monoterpenes in water and make them more effective [325].

### 3.17. Coleus

*Coleus*, another medicinal and aromatic genus of Lamiaceae [326,327], comprises 294 species found in the tropics and subtropics of Europe, Asia, Africa and Australia [115] (Table 1). The allelopathic properties of a few *Coleus* species have been reported (Table 2). Kathiresan [204] observed that a water suspension of *Coleus amboinicus* L. leaf dried powder at 40 g L^−1^ reduced the fresh and dry weight of *Echhornia crassipes* by 81 and 76%, respectively, within a week. The lowest dose required to kill *E. crassipes* was 10 g L^−1^. The extract was even injurious at 0.1 g L^−1^ when applied to cut leaves of *E. crassipes*. In another study, Gnanavel and Kathiresan [203] reported the allelopathic properties of *Coleus* spp. varied among their different parts. For example, dried leaf powder at 25 g L^−1^ of water was found to be most effective in reducing the fresh weight and chlorophyll content of *E. crassipes* and showed a 100% reduction on 9 and 6 days after treatment, respectively [203]. The second highest inhibitory activity was found when 3/4th of dried leaf powder at 18.75 g L^−1^ + 1/4th of dried whole plant powder at 6.25 g L^−1^ was applied. Interestingly, dried stem powder at 25 g L^−1^ showed a minimum reduction on those two parameters [203]. The aqueous leaf extracts of *Coleus forskohli* significantly inhibited the seed germination, root-shoot length and dry weight, and sugar and protein content of *Triticum aestivum*, while the opposite activity was found for total amino acid contents [205].

### 3.18. Calamintha

*Calamintha*, a small aromatic genus of Lamiaceae, is mainly distributed in the Mediterranean region [29,117]. The species of this genus also are traditionally used as folk medicines. Some *Calamintha* species are used for culinary purposes and herbal tea [117]. The allelopathic properties of *Calamintha* spp. with weeds and crop species under laboratory and field conditions have been reported in the literature (Table 2, Table 3 and Table 4).

Tanrisever et al. [328] isolated and identified menthofuran, calaminthone, terpenoids (+)-evodone, caryophyllene oxide, and ursolic acid from the aerial parts (through NMR and MS), and menthofuran and 2,3-dihydroevodone from the volatiles (through GC-MS) of *C. ashei*. The seed germination of *Schizachyrium scoparium* was strongly inhibited by the evodone and calaminthone volatiles. While, 100% inhibition on the seed germination of *S. scoparium* was observed when the seeds were subjected to a fraction of the aqueous solution containing calaminthone, evodone, and caryophyllene oxide. Interestingly, these fractions had no significant effects on *Lactuca sativa* seed germination. On the other hand, a stimulatory activity on *S. scoparium* was observed when a saturated aqueous solution of pure evodone was applied. In contrast, the opposite activity was found when evodone was applied together with the saturated aqueous solution of ursolic acid. Weidenhamer et al. [329] isolated and identified (+)-evodone and desacetylcalaminthone as the major constituents of *C. ashei* leaf soaks and washes through reversed-phase HPLC. They reported that an equimolar mixture of desacetylcalaminthone and (+)-evodone inhibited the germination of *Rudbeckia hirta* L. by 17% at a combined concentration of 0.025 mM, while the germination of *S. scoparium* (Mich x.) Nash cv. Cimarron and *Leptochloa dubia* (H.B.K.) Nees. was not affected below the concentrations of 0.125 and 0.25 mM, respectively. Besides the allelopathic properties of *Calamintha* spp., their species have insecticidal and antimicrobial properties. Božoviè and Ragno [117] reviewed the biological properties of *C. nepeta* (L.) Savi and its essential oils. The bioactive EOs constituent ‘pulegone’ of *C. nepeta* is considered as one of the three most toxic insecticides naturally occurring in many Lamiaceae species [273,330], because of the repellent, antifeeding, antidevelopment, and anti-reproduction behavior of pulegone for different harmful insects [331,332,333].

## 4. Knowledge Gaps and Future Prospects

In general, plants with medicinal properties are considered important sources of bioactive compounds [5]. Scientists are, therefore, showing interest in this category of plants for searching for novel bioactive compounds. The easier screening process for allelopathic plants and the possibility of having more bioactive compounds from medicinal plants than other plants are the two main reasons for this interest [142]. It is assumed that, due to the presence of many pharmacological, pharmaceuticals, and toxicological properties, Lamiaceae occupies 43% of the total studied species among the plant families examined for their bio-herbicidal potentialities [334]. Although this is a huge number compared to other plant families, there is a lot of empty space for researchers to work with the species of this family, to explore their allelopathic potential. However, the structural complexity, cost and time involvement in structure determination, labile characteristics of some compounds, and drawbacks in obtaining sufficient quantities for structure elucidation are amongst the major constraints for the isolation and characterization of allelopathic substances.

Beside these issues, most of the works were conducted in different laboratories with different setups, and thus it is rather difficult to compare the effectiveness of crude extracts, dried plant materials, or purified allelochemicals from different species as potential natural herbicides. Some purified allelochemicals were examined for their inhibitory activities on seed germination and/or the plant growth of crops and weeds, and they were far less active than commercial pesticides; although in most cases, commercial herbicides were not included as positive controls in the assays. The most effective allelochemicals appear to be volatile monoterpenoids, but some of these are also toxic to animals [13], and must, therefore, be used with caution. In fact, the challenges of finding greener herbicides have been discussed across fields [13,335,336]. If these limitations can be overcome, there is huge potential for using allelopathy/allelopathic substances of Lamiaceae plants in agriculture, as detailed below:

(i) ***Organ-specific:*** The allelopathic activities of some plants of this family are organ-specific. For example, the allelopathic activity of *N. meyeri* depended on whether the extract was derived from the leaves or roots, and maximum inhibitory activity was found with leaf extracts [135]. Similarly, dried leaf powder of *Coleus* spp. at 25 g L^−1^ of water significantly reduced the fresh weight and chlorophyll content of *E. crassipes*, while dried stem powder at the same concentration showed a minimal reduction on these two parameters [203].

(ii) ***Test plant-specific:*** It is assumed that the allelopathic plants or their allelochemicals that will be used for crop protection should be non-toxic or stimulatory to the crops. Nevertheless, in reality, this is not always true. However, many essential oils (EOs) of Lamiaceae species showed test plant-dependent inhibitory activity, where crops were less affected than weeds. For instance, linalool and linalyl acetate of *Lavandula* × *hybrida* showed phytotoxic activity against weeds (*Lolium rigidum* and *Phalaris brachystachys*) and no activity against crops (*Triticum aestivum* and *Hordeum vulgare*) [218]. The EOs of *Satureja hortensis* inhibited the germination of *A. retroflexus* at a lower concentration, while tomatoes were unaffected at the same concentrations. At the highest tested concentration, tomato germinations were affected, but less than *A. retroflexus* [34]. Similarly, when applying EOs of *Origanum onites* and *Rosmarinus officinalis* to the germination and seedling growth of *Avena sterilis*, *Sinapis arvensis*, and wheat cultivars, the wheat cultivars were less affected [37]. Kole et al. [313] reported a 45% reduction in weed population at 21 days after application but no effect on the *Triticum aestivum* growth by *Tectona grandis* fallen leaf powder in field conditions. In contrast, Lacret et al. [314] and Macías et al. [315] reported the germination and seedling growth inhibition of *Triticum aestivum* by EOs isolated from the dried leaves of *T. grandis* under laboratory conditions. The root and leaf extracts of *Nepeta meyeri* showed an allelopathic effect on the germination and seedling growth of barley and sunflower, while having no inhibitory activity on *Triticum aestivum* [135].

(iii) ***Phytotoxic under natural settings:*** Although thousands of species from different families or their isolated allelochemicals have been reported as allelopathic in the literature, most of them were laboratory experiments, and a few were greenhouse or field trials. A negligible number of reports are found in the literature where plant species showed allelopathic activity under natural settings. The Lamiaceae species, *Salvia leucophylla* and *Nepeta meyeri* belong to this short list that showed allelopathic activity under natural settings [119,209]. *Leucas aspera* and *Hyptis suaveolens* also form colonies under natural settings that suppress the growth of surrounding neighboring species, where allelopathy might play a vital role [30,32].

(iv) ***Presence of ursolic acid:*** Ursolic acid is present in some Lamiaceae species, which has a great role in their allelopathic activities. This ursolic acid is considered a natural detergent that makes the water-insoluble monoterpenes water-soluble and, consequently, boosts their ability to leach into rainwater for delivery into the soil [324].

(v) ***Terpenes as the major compounds:*** Terpenoids are the major group of plant specialized metabolites with allelopathic properties [181,337]. These compounds use IPP as a substrate which derives from either the MEP pathway [338] or the MVA pathway [339]. There are several types of terpenes, e.g., monoterpenes, sesquiterpenes, diterpenes, triterpenes, and polyterpenes [340]. Among them, monoterpenes (major elements of EOs) are most often reported to have herbicidal activity [181,334]. It is evident from this review that Lamiaceae species are rich sources of several allelopathic monoterpenes, e.g., camphor, α-pinene, β-pinene, 1,8-cineole, carveol, carvone, and camphene. Cinmethylin, a monoterpene-based commercial herbicide derived from 1,4-cineole, is already available on the market [341]. However, among the monoterpenes, the ketone-containing compounds, camphor and pulegone, are the most toxic, followed by alcohol compounds (cineol and citronellol), while ether, diene, and monoene compounds (α-pinene) are the least toxic [341,342]. In addition, pulegone is considered one of the three most toxic natural insecticides in the world [273,330].

(vi) ***Presence of volatile compounds:*** Although plant volatiles are considered an important tool for pest management in organic agriculture [343], unlike other mechanisms of allelopathy, very little research has been conducted to date to examine the effects of volatiles in plant–plant interactions [208]. It has been reported that plant volatiles mainly belong to the terpenoids, fatty acid derivatives, and phenolics groups [344]. As stated earlier, Lamiaceae species are rich sources of volatile EOs that are phytotoxic; for example, pulegone, α-pinene, limonene, 1,8-cineole, carvacrol, camphor, thymol, etc. [27,41,42,43,341,345]. Several plant-originated monoterpenoids are more toxic to nematodes than commercial nematicides. Compounds such as thymol and carvacrol have been found to be the most effective, with 100% mortality [346]. Furthermore, eugenol, geraniol, isoeugenol, and methyl isoeugenol also have nematicidal properties [347].

(vii) ***Scalable by biotechnology:*** Traditional breeding has been adopted for Lamiaceae species, especially the ones that are an important source of essential oils, such as mint [348,349] and catnip [350,351]. The recent findings in the botany and horticulture of catnip have been reviewed, and the interest in scaling up catnip to industrial scale was also discussed in Gomes et al. [352]. This evidence supports our view of further utilizing and exploring Lamiaceae in sustainable agriculture. With the recent advance in sequencing technologies, the genomes of many Lamiaceae species have been sequenced. This includes species in Salvia [353,354], Nepeta [355], Lavendula [356], Ocimum [357], and Mentha [358], whose allelopathy was described above. In addition, more transcriptomes are available on several platforms, such as the mint genome project (http://mints.uga.edu, accessed on 13 January 2022) and OneKP [359]. The availability of omics data allows scientists to gain fundamental knowledge of terpene biosynthesis and adopt biotechnology tools to increase the yield and productivity of the targeted compounds. Many synthetic biological and metabolic engineering approaches have successfully created production platforms for terpenoids. Zebec et al. [360], and more recently Zhang and Hong [361], have comprehensively reviewed the synthetic biology approach for monoterpene production in *Escherichia coli* and *Saccharomyces cerevisiae*, the industrial workhorses. The metabolic engineering attempts and significant milestones of terpenoid production in planta were also discussed by Mani et al. [362]. To date, the cell-free enzyme system from *E. coli* has shown the highest yield of monoterpene production, which is up to 14–15 g L^−1^ [363]. The fed-batch fermentation system from *E. coli*, which is more feasible on an industrial scale, can produce limonene up to 3.6 g L^−1^ [364]. Therefore, it is highly likely that the allelopathic compounds of interest in Lamiaceae can be synthesized on an industrial scale, by taking advantage of the recent discoveries in genetics, breeding, biochemistry, and synthetic biology. This advantage of these biotechnological tools may help overcome the challenges in field testing, by making the allelopathic compounds more accessible.

These special features of allelopathic plants from Lamiaceae families, or their isolated allelochemicals, may help researchers develop natural product-based crop or weed specific herbicides and insecticides. Although the utilization of allelopathy in agriculture could be achieved with the incorporation of allelopathic plant residues into soils or with mulching, the application of water extracts or essential oils from allelopathic plants, and allelochemicals produced artificially (organic synthesis, fermentation, tissue culture, etc.) [5], and extraction with organic solvents should be avoided for green allelopathy. Therefore, a huge amount of research with allelopathic Lamiaceae plant species remains to be conducted, both under controlled laboratory and field conditions, to harness their maximum potential for agricultural purposes. A significant amount of research is also needed to explore the mechanisms of action, impacts on beneficial crops, insects, and other organisms, formulations for effective application, and to assess the cost–benefits of allelochemicals identified from Lamiaceae species.

## 5. Conclusions

Researchers have always had a keen interest in the Lamiaceae plant species because of their multitude of pharmacological and pharmaceutical properties and the presence of bioactive EOs. However, considering the number of plant species, a significant amount of research is yet to be conducted, to explore the allelopathic activity of the Lamiaceae family. As for other plant families, most of the research works carried out to date have mainly focused on the inhibitory properties of Lamiaceae plant extracts and their EOs on the germination and seedling growth of several target species, under controlled laboratory or greenhouse conditions. Therefore, the transfer of laboratory and greenhouse experiments into field settings is imperative, for understanding the environmental impacts on the herbicidal/pesticidal activity of the allelochemicals of Lamiaceae plants or their EOs on target species. In addition, the biostimulatory activities of allelochemicals/EOs at lower concentrations should also be given more priority. With this review, we provide the current stage of the research in allelopathy and point out the potential of allelopathy in Lamiaceae species as a source of greener alternative herbicides, along with the gaps in knowledge that need filling for introducing natural allelopathic substances to agriculture.

## Figures and Tables

**Table 1:** List of major genus and species of the Lamiaceae family, with their distribution and special characteristics.

Sl. No.	Genus Name	Total No. of Species	Distribution	Chemical Constituents *	Medicinal and/or Industrial Properties	Reference
1	*Salvia*	900	Throughout the Old world (Asia, Africa and Europe) and new world (Americas)	Sesquiterpenoids, diterpenoids, sesterterpenoids, triterpenoids, steroids, polyphenols, etc.	Antioxidative, antibacterial, hypoglycaemic, anti-inflammatory, fungistatic, virustatic, astringent, eupeptic, anti-hydrotic, and cardioprotective properties. Used as spices and flavoring agents.	[44,45,46,47]
2	*Nepeta*	280	Native to temperate Europe, Asia, Africa, and are naturalized in North America	Nepetalactone (and its isomers), 1,8-cineole, β-caryophyllene, caryophyllene oxide, β-farnesene, α-citral, β-citronellol	Diuretic, diaphoretic, antitussive, antispasmodic, antiasthmatic, febrifuge, emmenagogue, sedative, antitumor, anti-inflammatory, antimicrobial, feline and canine attractant, insect repellant, arthropod defense, antibacterial, antifungal, and antiviral properties. Used as a perfume and flavoring agents.	[48,49,50,51,52,53]
3	*Ocimum*	160	Widely distributed throughout the tropical and sub-tropical Asia	Eugenol, thymol or sesquiterpene alcohols as major or terpene compounds as minor oil constituents	Anti-diabetic, anti-oxidant, anti-microbial, antinociceptive, anti-fertility, anti-inflammatory, anti-cancer, anthelmintic, cardioprotective, etc.	[54,55,56]
4	*Lavandula*	30	Native to the Mediterranean region, but is grown in many other countries of the world	Linalool, linalyl acetate, 1,8-cineole β-ocimene, terpinen-4-ol, and camphor	Anticancer, antimutagenic, antioxidant, antimicrobial, anxiolytic, mood stabilizer, sedative, analgesic, anticonvulsive and neuroprotective properties. Used for the treatment of epilepsy, migraine attacks, pain and tremor. Also used in perfume, cosmetic industry, and aromatherapy.	[57,58,59,60]
5	*Mentha*	42	Northeastern Africa, western Asia and southeastern Europe	Menthol	Insecticidal, antibacterial, antifungal, anti-cancer, pharmaceutical, flavoring and cosmetic properties. Used for treating wounds, swollen glands, cough, cold, fever, asthma, indigestion, influenza, vomiting, gastro-intestinal disorder.	[61,62,63,64,65,66,67,68]
6	*Thymus*	400	Native to Europe	Several types of monoterpenes, p-cymene, γ-terpinene and thymol	Antiseptic, antihelminthic, expectorant, antispasmodic, antimicrobial, antifungal, antiviral, antioxidative, carminative, sedative, antivirotic, diaphoretic, antibacterial, antispasmodic, antirheumatic, antihypertensive, anti-inflammatory, and pharmaceutical properties. Used for the treatment of skin (oily skin, acne, dermatitis), eczema, insect bites, digestive, cardiovascular, nervous systems, nausea and fatigue, respiratory (such as colds), menstrual and menopausal problems, etc.	[69,70,71,72,73,74]
7	*Hyptis*	150	Tropical America, but now distributed throughout the world from tropical to subtropical regions.	Urosolic acid, alkaloids, terpenes, and volatile oils	Natural HIV-integrase inhibitor, antispasmodic, antirheumatic, anti-inflammatory, antifertility agents, antiseptic, appetizer and insecticidal properties. Used for diabetes and cancer treatments.	[32,75,76,77,78]
8	*Leucas*	80	Tropical and temperate Asia, and Africa	Lignans, flavonoids, coumarins, steroids, terpenes, fatty acids, and aliphatic long-chain compounds	Analgesic, antipyretic, anti-rheumatic, anti-venom, anti-inflammatory, antibacterial, antifungal, and mosquito repellent properties. Used for coughs, colds, painful swellings, and chronic skin eruption treatment.	[25,79,80]
9	*Leonurus*	20	Europe and Asia, naturalized in New Zealand, Hawaii, New Caledonia, and America	Diterpenoids	Analgesic, anti-inflammatory, anti-bacterial, antiproliferative, antioxidative, anticancer, cardioprotective, neuroprotective properties. Used for treating chronic rheumatism, menstrual irregularities, and heart disorders.	[22,81,82,83,84,85]
10	*Origanum*	900	Europe, Central Asia, and North America	Carvacrol, Thymol, γ-terpinene, p-cymene, β-cymene, Methyleugenol, myristicin	Anti-fungal, anti-bacterial, anti-tumor, anti-inflammatory, anti-oxidant, anti-cholinesterase, anti-parasitic, anti-viral, and anti-diabetes properties.	[86,87,88]
11	*Rosmarinus*	3	Native to the Mediterranean areas, and widely distributed in many parts of the world	α-pinene, verbenol, verbenone, 1,8-cineol and isoborneol	Antimicrobial, antioxidant, antibacterial, antimycotic, food flavoring, and food preservative properties.	[89,90,91]
12	*Hyssopus*	36	Highly abundant on dry, rocky, calcareous soils in Europe, southwest and central Asia, and north-west India	Pinocamphone, α-pinene, β-pinene, apigenin, quercetin, diosmin, luteolin, chlorogenic, protocatechuic, ferulic, syringic, p-hydroxybenzoic, and caffeic acids	Used for the treatment of stomachic, chronic bronchitis, rheumaticpains, bruises, wounds, blood pressure regulation, states of anxiety, hysteria. Has muscle-relaxing, antiseptic, insecticidal, nematicidal, antibacterial, antifungal, and antioxidant properties.	[92,93,94,95,96,97]
13	*Orthosiphon*	40	Tropical and subtropical Asia including Southern Africa and Madagascar	Polymethoxylated flavonoids, phenylpropanoids (caffeic acid derivatives), and terpenoids (mainly diterpenes and triterpenes)	Used for the treatment of urinary lithiasis, edema, rheumatism, hepatitis, diabetes, hypertension, oedema, epilepsy, fever, influenza, tonsillitis, menstrual, disorder, gonorrhea, syphilis, and jaundice.	[98,99,100,101]
14	*Tectona*	3	Indian subcontinent, throughout Myanmar and Thailand	Triterpenoids, flavonoïds, chromomoric acid derivatives, anthraquinones, naphthoquinones, anthraquinone-naphthoquinones, apocarotenoids and lignans	Used for bronchitis treatments, hyperacidity, dysentery, verminosis, burning sensation, diabetes, difficult labor, leprosy, skin diseases, stomatitis, indolent ulcers, headache, biliousness, burning pains, etc. Have hemostatic, anti-inflammatory, antibacterial, antifungal, analgesic, cytotoxic, hypoglycemic properties.	[40,102,103,104]
15	*Satureja*	200	Mediterranean region, Asia, and some parts of USA	Thymol, carvacrol, cymene, flavonoids, tannins, linalool, γ-terpinene	Antimicrobial, antioxidant, anti-inflammatory, anti-parasitic, anti-viral, analgesic, antinociceptive, anti-diabetic, anti-cancer, and anti-hypercholesterolemic properties.	[105,106,107]
16	*Conradina*	6	Xeric with well-drained sandy soil areas of US	Camphor, 1,8-cineole, ursolic acid, cis-punocamphone, botulin, α-pinene, p-pinene, myrtenal, myrtenol, verbenone, myrtenyl acetate, limonene, camphene, β-amyrin, β-caryophyllene, β-pinene, β-cubebene, myrtenic acid	Antibacterial, antifungal, cytotoxic, antileishmanial properties.	[108,109]
17	*Coleus*	264	Tropics and sub-tropics of Old world	Terpinolene, α-pinene, β-pinene, β-caryophyllene, 1, 8-cineole, eugenol, carvacrol, thymol and β-phellandrene	Stimulant, antispasmodic and stomachic properties, and used for the treatment of headache, fever, epilepsy, dyspepsia, chronic cough, and asthma	[110,111,112,113,114,115,116]
18	*Calamintha*	9	Mediterranean region	Pulegone, menthone isolmenthone, piperitone, carvone, gallic acid, rosmarinic acid, caffeine, caffeic acid and eucalyptol	Antimicrobial, antiseptic, antispasmodic, antimicrobial, antispasmodic, sedative, and antipyretic properties.	[117,118]

* Major compounds found in most of the species of the respective genus.

**Table 2:** Allelopathic activity of Lamiaceae plant species under laboratory conditions.

Plant Species	Plant Organ	Extract Types	Target Species	Effect	Reference
*Salvia moorcraftiana* Wall.	Aerial parts	Crude acetone extract	*Lemna aequinoctials* Welve.	Inhibited growth	[129]
*Salvia sclarea*	Aerial parts	Aqueous extract	*Solanum nigrum* L. roots	Induced lipid peroxidation	[130]
			*Bromus mollis* L.	Increase of the superoxide dismutase, catalase, and antioxidant enzyme activity	[131]
*Salvia macrosiphon* Boiss.	Aerial parts	Aqueous extract	*Zea mays* L.	Inhibited seed germination, growth, fresh, and dry weight of radicles and plumules	[132]
*Salvia macrochlamys* Boiss. et Kotschy	Aerial parts	Methanolic extract	*Portulaca oleracea*	Inhibited the germination, decreased the amylase activity and the abscisic acid (ABA) at higher concentrations (>2.5%), increased the gibberellic acid (GA_3_) levels at conc. <2.5%	[124]
*Salvia officinalis* L.	Aerial parts	Aqueous extracts	*Hordeum vulgare* and *Portulaca oleracea*	Inhibited germination	[133]
		Essential oils (EOs)	*Lectuca sativa*	Inhibited germination and root growth	[46]
*Salvia namaensis* Schinz, *Salvia fallax* Fernald, *Salvia disermas* L., *Salvia chamaedryoides* Cav., *Salvia confertiflora* Pohl., *S*. × *jamensis* J. Compton, *Salvia buchananii* Hedge, *S. wagneriana* Polak, *Salvia scabra* Linn. Fil., *Salvia miniata* Fernald, *Salvia cacaliaefolia* Benth., *Salvia adenophora* Fernald, *Salvia rutilans* Carrière	Aerial parts	Exudate	*Papaver rhoeas* L. and *Avena sativa* L.	Inhibited germination and growth	[122]
*Nepeta nuda* subsp. Nuda	Aerial parts	Water extracts	*Cucumis sativus* L. and *Triticum aestivum* L.	Seedling growth, fresh and dry weight	[134]
*Nepeta meyeri* Benth.	Roots and leaves	Aqueous extracts	*H. vulgare*, *T. aestivum*, *Brassica* napus L., *Carthamus tinctorius* L. and *Helianthus annuus* L.	Inhibited seed germination and seedling growth of *H. vulgare* and *H. annuus* at all concentrations. Stimulated *T. aestivum B. napus* and *C. tinctorious* seedling growth at lower concentrations. At higher concentration showed neutral activity to *T. aestivum*, but inhibited *B. napus* and *C. tinctorious*.	[135]
*Nepeta preatervisa*	Whole plants	Methanolic extract	*Lemna aequinocatialis*	Inhibited the development of fronds	[136]
*Ocimum basilicum* L.	Leaf, root and seeds	Aqueous extracts	*T. aestivum*, *Cicer arietinum*, *Lens culinaris*, *Brassica* spp., *Hordeum vulgare*, *Abelmoschus esculentus*, and *Pisum sativum*	Inhibited seed germination and seedling growth	[137]
	Aerial parts	Aqueous extract	*Sorghum bicolor* [L.] Moench, *Pennisetum glaucum* [L.] R. Br., *Zea mays* L., *Triticum vulgare* L.		[138]
		Crude methanolic extracts	*Lemna minor*	Moderate phytotoxicity (25%) was obtained at 1000 µg mL^−1^ concentration	[139]
		Methanol, acetone and distilled water	*Z. mays* and *Glycine max*	Root growth	[140]
		Aqueous extract	*Sorghum bicolor* [L.] Moench, *Pennisetum glaucum* [L.] R. Br., *Z. mays* and *Triticum vulgare* L.	Inhibited the seed germination of the tested cereal crops and the order of their sensitivity was *Z. mays* > *P. glaucum* t > *T. vulgare* > *S. bicolor*	[141]
*Ocimum tenuiflorum* L.	Whole plants	Aqueous methanol extract	*Lepidium sativum* L., *Lactuca sativa* L., *Medicago sativa* L., *Lolium multiflorum* Lam., *Echinochloa crus-galli* L. and *Phleum pratense* L.	Inhibited the total germination percentage, germination index, germination energy, speed of emergence, seedling vigor index, coefficient of the germination rate, except those for *E. crusgalli* and germination % of *L. sativa* at higher concentration. Increased the time required for 50% germination and mean germination time.	[142]
*Ocimum sanctum* L.	Dry leaf extract	Aqueous extract	*Phaseolus radiata* (L.) Wilczek, *Phaseolus unguiculata* (L.) Walp, *Cajanus cajan* L., *Cicer arietinum* L., *Phaseolus mung*o (L.) Heeper, and *Phaseolus aconitifolius* Jacq.	No inhibition on the seed germination of the legumes, except for *C. arietinum*	[143]
	Leaf extract		*Amaranthus spinosus* L.	Inhibited seed germination (80%)	[143]
*Lavandula officinalis*	Dry leaf extract	Aqueous extract	Velvet flower and Purslane	Reduction of germination, stem and root growth, and fresh weight	[144]
*Lavandula* × *intermedia*	Dry flowers	Aqueous extract	*L. sativa*	Inhibited germination, seedling length, and fresh and dry weight of seedlings	[145]
*Lavandula* × *intermedia* cv. Grosso	Leaf and stem	Aqueous extract	*Lolium rigidum*	Completely inhibited root growth	[146]
*Lavandula* × *intermedia Emeric ex* Loisel.	Flowers	Aqueous extract	*R. sativus*	Completely inhibited seed germination	[147]
*Mentha* × *piperita* L.	Aerial parts	Aqueous extract	*R. sativus*	Negative effect on germination, growth, and super oxide dismutase, and positive effect on proline, soluble sugars and total phenols, ascorbate peroxidase, catalase, and peroxidase	[148]
*Mentha longifolia* syn. *M. sylvestris* L.	All parts of the plant (leaves, stem, flowers, seeds and roots)	Methanolic extract	*T. aestivum*	Inhibited shoot and root growth	[66]
*Mentha sylvestris* L.	All parts of the plant (leaves, stem and roots)	Aqueous methanolic extract	*Lepidum sativum* L., *L. sativa* L., *Medicago sativa* L., *B. napus*, *Phleum pretense* L.; *Digitaria sanguinalis* L. scop.; *E. crus-galli*, and *L. multiflorum*	Inhibited seedling growth	[149]
*Mentha* × *piperita* L.	Dry leaves	Volatile compounds	*R. sativus*	Inhibited germination	[150]
*Mentha longifolia* L.	Dry leaves	EOs	*Convolvulus arvensis* L.	Inhibited seed germination, and root and shoot growth	[151]
*Mentha* × *villosa* Huds.	Soil collected from the garden area cultivated with mint	-	*L. sativa*	Inhibited seed emergence, but had no effect on germination speed index	[152]
*Mentha spicata* L.	Foliage	EOs	*Alcea pallida* Waldst. and Kit., *Amaranthus retroflexus* L., *Centaurea salsotitialis* L., *R. raphanistrum*, *Rumex nepalensis Spreng*., *Sinapis arvensis* L., and *Sonchus oleraceus*	Inhibited seed germination	[153]
*Thymus serpyllum*	Fresh aerial parts	Methanolic extract	*Lemna minor* L. and *R. sativus*	Inhibited germination and growth	[154]
*Thymus numidicus Poir*.	Leaves, stem and roots	Water, petroleum ether, ethyl acetate and methano	*Medicago sativa* and *T. aestivum*	Inhibited germination and growth	[155]
*Thymus kotschyanus*	Whole plants	Aqueous extracts	*Bromus tomentellus* and *Trifolium repens*	Inhibited germination and seedling growth, and fresh and dry weight	[156]
*Thymus vulgaris*	Leaves	Aqueous extracts	*C. arietinum*	Reduced germination capability, shoot and root length, total free amino acids, and proline content. Whereas, increased the carbohydrates, proteins, K^+^, Ca^2+^, and the activity of antioxidant enzymes	[157]
*Thymus comosus Heuff. ex Griseb*. & *Schenk*, *Thymus dacicus Borbás* and *Thymus praecox* ssp. *polytrichus* (*A. Kern. ex Borbás*) *Jalas*	Aerial parts	Aqueous extracts	*R. sativus* and *B. oleracea*	Reduced germination percentage, speed of germination, and accumulated speed of germination	[158]
*Thymus vulgaris*	Soil under *Thymus* plants	-	*Daucus carota*, *Nigella damascena*, and *Bromus madritensis*	Reduced germination	[159]
*Hyptis rhomboide* Mart. et Gal	Stalks	Aqueous extracts	*B. campestris*, *R. sativus* and *O. sativa*	Inhibited the seed germination and seedling growth of *Brassica campestris*, *Raphanus sativus* L., *Oryza sativa*	[160]
	Leaves				
*Hyptis suaveolens* (L.) Poit.	Leaves	Leaves residue	*S. vulgare* and L. *sativa*	Reduced germination speed index and percentage of germination	[161]
		Aqueous extracts	*C. arietinum* and *C. cajan*	Fungal infections on seeds were observed after 8 days	[162]
		Leaf leachates	*Parthenium hysterophorus*, *Senna uniflora*	Inhibiting seed germination	[163]
		Leaf extracts and leachates	*Vigna radiata* cv. *K851*	Reduced the germination, seed viability, insoluble carbohydrates, proteins, and the activities of dehydrogenase and catalase enzymes. Increased the amino acid and soluble carbohydrate levels.	[164]
		Aqueous extracts	*T. aestivum var k9*	Reduced germination percentage, weight of germinated seeds, radicle and coleoptile length, total chlorophyll, and total proteins	[165]
		Ethanolic extract	*L. sativa*, *G. max*	Strong inhibitory activity was observed on the germination percentage, germination speed index, growth inhibition of seedlings and biomass production of *L. sativa*	[166]
		Aqueous extracts	*O. sativa*	Decreased the percentage of germination	[167]
*Hyptis suaveolens* (L.) Poit	Whole plants	Aqueous methanolic extracts	*Lepidum sativum* L., *L. sativa*, *M. sativa*, *B. napus*, *Phleum pratense* L., *Digitaria sanguinalis* L. Scop., *E. crus-galli*, and *L. multiflorum*	Inhibited the germination of *L. sativum* and *L. multiflorum*, and the seedling growth of all test species	[78]
*Hyptis sauveolens* (L.)	Leaves and roots	Aqueous extracts	*O. sativa* cv. *Gobindobhog*	Inhibited germination, shoot and root length	[168]
*Leucas aspera* (Willd.) Linn.	Whole plants	Aqueous methanolic extracts	*L. sativum* L., *L. sativa*, *M. sativa*, *P. pratense*, *E. colonum*, *E. crus-galli*, *and L. multiflorum*	Inhibited the seedling growth of all test species	[169]
*Leucas cephalotes* (Roth)	Leaves and roots	Aqueous extracts	*O. sativa* cv. Gobindobhog	Inhibited germination, shoot and root length	[168]
*Leonurus sibiricus* L.	Aerial parts	Aqueous extracts	*T. aestivum*	Inhibited seed germination and seedling growth	[170]
	Whole plants	Aqueous methanolic extracts	*L. multiflorum*, L. *sativum*, and *L. sativa*	Inhibited seed germination	[171]
			*L. multiflorum*, L. *sativum*, *L. sativa*, *P. pratense*, *D. sanguinalis*, *M. sativa*, and *B. napus*	Inhibited seedling growth	
	Aerial parts	Aqueous, ethanol, and acetone extracts	*Solanum melongena*, *Abelmoschus esculentus*, *Amaranthus tricolor and Cucumber Cucumis sativus*	Inhibited seed germination and seedling growth	[172]
*Hyssopus officinalis* L.	Aerial parts	Water infusions	*Cucumis sativus* L. and *T. aestivum*	Inhibitory effects on germination and root elongation (*T. aestivum > C. sativus*)	[173]
			*Allium cepa* L.	Mitodepressive and genotoxic effect on the root tip cells	
*Origanum vulgare* ssp. hirtum (Link)	-	EOs	*Arabidopsis seedlings*	Inhibited glutamate and aspartate metabolism, altering the photorespiratory pathway	[174]
*Origanum onites* L.	-	EOs	*T. aestivum*, *Avena Sterilis* and *Sinapis arvensis*	Inhibited germination and seedling length	[37]
	-	Volatile oils	*Onobrychis viciifolia*	Reduced plant length	[175]
*Origanum vulgare ssp. vulgare* L.	Aerial parts	Cold water extracts	*T. aestivum*	Decreased root length	[176]
			*Allium cepa* L.	Inhibited cell division in root meristematic cells, induced abnormalities in mitotic and interphase cells	
			*Cucumis sativus* L.	Decreased root length	[177]
*Origanum majorana* L.	Seed, aerial parts	Co-germination, Aqueous extracts	*Z. mays*	Co-germination stimulated *Z. may*s germination, whereas aqueous extracts inhibited root length	[178]
*Origanum compactum Benth*.	Leaves	Aqueous extracts	*Microcystis aeruginosa*	Inhibited the growth and decreased the photosynthetic pigments (chlorophyll-a and carotenoids)	[179]
*Rosmarinus officinalis* L.	Dry plant powder	EOs	*M. aeruginosa* and *Chroococcus minor*	Decreased growth rates	[180]
	Aerial parts	EOs	*L. sativa*, *A. retroflexus*, *P. oleracea*, and *Acroptilon repens*	Inhibited seed germination and growth	[181]
	Aerial parts (inflorescences, leaves and stems)	Solid residue	*Lycopersicon esculentum* L. and *Lolium perenne* L.	Limited phytotoxic effects on germination, root and leaf growth	[182]
	Leaves	Aqueous extracts	*Panicum turgidum Forssk*.	Inhibited germination percentage, relative germination percentage, plumule and radicle lengths	[183]
	Aerial parts	EOs	*Cynodon dactylon* L., *Festuca arundinacea* Schreb. and *Lolium perenne* L.	Inhibited seed germination and growth	[184]
*Satureja montana* L.	Aerial parts	Aqueous extracts	*Capsicum annuum* L. and *Solanum nigrum* L.	Not phytotoxic, induced lipid peroxidation in *S. nigrum* roots, and increased the pyrogallol and guaiacol peroxidase in *S. nigrum* leaves	[185]
*Satureja thymbra* L.	Aerial parts	Aqueous extracts	*Pinus halepensis Mill*. and *Ceratonia siliqua* L.	Inhibited the germination of *C. siliqua*, and the root length and number of leaves of *P. halepensis* and *C. siliqua*	[186]
*Satureja montana* L.	Aerial parts	Aqueous extracts	*Datura stramonium* L.	Induced lipid peroxidation in roots of *D. stramonium*	[187]
			*G. max*	Increase in the catalase and superoxide dismutase activity of roots, and the superoxide dismutase activity in leaves	[188]
*Satureja khuzestanica* Jamzad, *S. bachtiarica* Bunge and *S. rechingeri* Jamzad	Aerial parts	Aqueous extracts	*L. sativum*, *Solanum lycopersicum*, and *Secale cereale*	*S. khuzestanica* aqueous extract was most suppressive to *S. cereale* seed germination, while *S. bachtiarica* aqueous extract suppressed the germination and growth indices of *L. sativum*, *Solanum lycopersicum* and seedlings	[189]
	Leaves	Dry leaves powder		*S. rechingeri* had the maximum inhibitory effect on germination percent and growth indices of *S. cereale*, L. *sativum* and *Solanum lycopersicum*	
*Satureja hortensison* L.	Aerial parts	Aqueous extracts	*P. oleraceae* and *Chenopodium album*	Inhibited the root, stem, leaf growth, root/shoot ratio, germination rate, and percentage germination	[190]
*Tectona grandis* L.	Green and deciduous leaves	Methanol extract	*E. colona*, *Cyperus difformis* L. and *O. sativa*	Inhibitory activity on *E. colona* germination, no activity on *O. sativa*	[191]
		Aqueous extracts		Inhibitory activity on *C. difformis* germination, no activity on *O. sativa*	
	Dried leaves	Aqueous extracts	*Vigna mungo var. ADT-3* and *V. radiate var. Co-3*	Completely inhibited the seedling growth, dry weight at 100% concentration	[192,193,194]
	Fresh leaves	Aqueous extracts	*Plumbago zeylanica Linn*.	Inhibited the seed germination and seedling growth	[195]
	Leaves and flowers	Aqueous extracts	*L. sativa*	No inhibitory potential on the percentage and average germination time	[196]
	Leaves	Leachates	*Vigna unguiculata*, *Momordica charantia* and *Solanum melongena* L.	Inhibited the seed germination and seedling growth	[197]
	Leaves	Aqueous extracts	*Vigna mungo* (L.) *Hepper*	Inhibited the seed germination and seedling growth	[198]
	Top soil	Aqueous extracts	*L. esculentum*	Suppressed germination and growth	[199]
*Orthosiphon stamineus Benth*. (*syn. O. aristatus*, *O. gradiflorus*, *O. spicatus*)	Shoots	Aqueous methanol extracts	*L. sativum* and *L. sativa*	Inhibited root and hypocotyl growth	[200]
*Calamintha nepeta* L. (Savi)	Leaves and stems	Methanol extract was further fractionated using n-hexane, chloroform, ethyl acetate and n-butanol	*L. sativa*	Inhibited germination and root growth of *L. sativa* with a methanolic extract and also with its fraction. Hierarchy of phytotoxicity of its fraction was ethyl acetate > n-hexane > chloroform > n-butanol	[29]
	Foliar	Volatiles and EOs	*L. sativa*, *R. sativus* and *A. retroflexus*	Volatiles strongly inhibited both germination and root growth of *L. sativa*, and EOs at >125 µL L^−1^ inhibited both processes of *L. sativa*, *R. sativus*, *A. retroflexus*	[201]
	Leaves and green stem	Aqueous extract	*L. sativa*, *C. album*, *Sinapis alba*,	Inhibited germination and root growth	[202]
*Coleus amboinicus* L.	Dried leaves powder	Aqueous extract	*Eichhornia crassipes* Mart.	Reduced the fresh and dry weight	[203,204]
*Coleus forskohli*	Leaves	Aqueous extract	*T. aestivum*	Root–shoot length and dry weight	[205]

**Table 3:** Allelochemicals isolated from Lamiaceae plant species and their allelopathic potential.

Plant Species	Allelochemical/Major Compounds	Parts from Where Isolated	Target Species	Effect	Reference
*Salvia miniata* Fernald	13 clerodane diterpenoids	Extracts of aerial parts	*Papaver rhoeas* L. and *Avena sativa* L.	Inhibited germination and growth	[125]
*Salvia elegans* Vahl, *Salvia greggii* A. Gray, *Salvia munzii* Epling	monoterpenoids and sesquiterpenoids	Essential oils (EOs)	*R. sativus*. and *L. sativum*	Inhibited germination and root growth	[126]
*Salvia leucophylla*	volatile monoterpenoids (camphor, 1,8-cineole, β-pinene, α-pinene, and camphene)	Volatile compounds from seeds	*B. campestris*	All five monoterpenoids inhibited root growth but camphor, 1,8-cineole, and β-pinene only inhibited germination at high concentrations	[127]
*Salvia miltiorrhiza*	neo-przewaquinone *A*	Roots	*M. aeruginosa*	Caused cell morphologic damage or lysis, increased malondialdehyde content, and decreased the soluble protein content, total antioxidant, and superoxide dismutase activity, and significantly inhibited three photosynthesis-related genes (psaB, psbD, and rbcL)	[206]
*Salvia broussonetii*	demethylsalvicanol and 14-deoxycoleon *U*	Roots	*Leptinotarsa decemlineata*	Antifeedant	[207]
	demethylcryptojaponol			Toxic	
*Nepeta faassenii*	2-(2-ethoxyethoxy)ethanol, alloaromadendrene, and Χ-cadinene	Volatile mixture and the methanolic extract, but also in an aqueous foliar extract	*L. sativum*	Growth	[208]
*Nepeta meyeri* Benth.	4aα,7α,7aβ-nepetalactone (83.4%)	EOs	*A. retroflexus*, *Bromus danthoniae* Trin., *Bromus intermedius* Guss., *L. serriola*, *C. album*, *C. dactylon*	Inhibited germination and seedling growth. Increased CAT activity in all the weed species, and decreased SOD activity, except in *A. retroflexus*. Also increased the lipid peroxidation and hydrogen peroxide (H_2_O_2_) concentration	[209,210]
	4aα,7α,7aβ-nepetalactone (80.3% in essential oils), 4aα,7α,7aβ-nepetalactone (83.7% in hexane extract)	Aerial parts	*A. retroflexus*, *C. album*, *Cirsium arvense* L. and *Sinapsis arvensis* L.	The essential oils completely inhibited the germination of all species. Concentration-dependent inhibitory activity by the extract.	[35]
*Nepeta curviflora* Boiss., *Nepeta nuda* L. subsp. albiflora (Boiss.) Gams., *Nepeta nuda* L. subsp. albiflora	Aerial parts	EOs	*R. sativus* and *L. sativum*	Germination and initial radical elongation	[211]
*Calamintha nepeta* (L.) Savi	-	Foliar volatiles	*L. sativa*	Inhibited both germination and root growth	[29]
	pulegone	EOs	*L. sativa*, *R. sativus* and *A. retroflexus*		
*Nepeta meyeri* Benth.	4aα,7α,7aβ-nepetalactone (80.4% in essential oils),	Aerial parts & EOs	*Bromus danthoniae*, *L. serriola*, *B. napus* and *Z. mays*	Inhibited the germination	[212]
*Nepeta flavida*	linalool (37.64%) and 1,8-cineole (30.80%)	Aerial parts EOs	*L. sativum*, *R. sativus* and *Eruca sativa*	Completely inhibited the germination at 4.0 and 8.0 μL mL^−1^	[213]
*Nepeta pannonica* L.	1,8-cineole (28.9%), and 4aα,7β,7aα-nepetalactone (14.3%)	Aerial parts EOs	*Agrostis stolonifera* cv. *Pencross*	100% growth inhibition at 0.3 mg mL^−1^	[214]
			*L. sativa* cv. *Iceberg*	100% growth inhibition at 1.0 mg mL^−1^	
*Ocimum americanum*	limonene, camphor and linalol	EOs	*Mimosa pudica* and *Senna obtusifolia*	Inhibited the germination and seedling growth	[215]
*Ocimum gratissimum*	flavonoids	Dried powdered leaves	Bean and *Z. mays* seedlings	Inhibited the radicles more than their coleoptiles	[216]
*Lavandula angustifolia* Mill.	lavandulol, terpinen-4-ol, linalyl acetate, lavandulyl acetate and α-terpineol	EOs	*Setaria verticillata* (L.) P. Beauv.	Inhibited germination and root length	[217]
*Lavandula* × *hybrida* Rev.	linalool (27.51%) and linalyl acetate (37.21%)	EOs	*Crops: T. aestivum* and *H. vulgare* and *Weeds: Lolium rigidum* L. and *Phalaris brachystachys* L.	Inhibited the germination and root length of weeds and had no effect on crops	[218]
*Lavandula* × *intermedia* cv. Grosso	coumarin and 7-methoxycoumarin	Leaf and stem extract	*L. rigidum*	Inhibited growth	[146]
*Lavandula stoechas*	stoechanones A and B	Aqueous methanol extract	*A. retroflexus*	Inhibited the seed germination percentage, radicle, and hypocotyl lengths	[219]
*Mentha longifolia* (L.) Huds.	piperitone oxide (53.83%) and piperitenone oxide (11.52%), followed by thymol (5.80%), and (*E*)-caryophyllene (4.88%)	EOs	*C. rotundus*, *E. crus-galli* and *O. sativa*	In a pre-emergence assay: Inhibiting percent germination, plantlet growth, and chlorophyll content of the weeds. In pre-emergence assay: loss of chlorophyll, wilting, and growth inhibition, leading to death of all species	[220]
			*Allium cepa* L. *root tips*.	EO exposure to the onion roots induced various chromosomal aberrations	
*Mentha spicata* L.	carvone (15.3–68.5%), piperetenone oxide (24.0–79.2%) and α-humulene (0.1–29.9%)	EOs	*S. tuberosum*	Sprout suppressant	[221]
*Mentha* × *piperita* L.	(-)-menthol (58.7–71.2%), menthone (3.5–19.6%), limonene (3.4–8.4%), menthyl acetate (1.4–17.2%) and β-caryophyllene (2.4–6.3%)	EOs	*R. sativus*	Stimulated the germination	[222]
*Mentha pulegium* L.	pulegone (57.8–62.8%), menthone (9.5–15.0%) and limonene (4.9–6.9%)	EOs	*M. sativa*	Inhibited the germination	[223]
*Mentha spicata* L., *M. crispa*, *M. longifolia*,	menthone/isomenthone	EOs	*L. sativum*	Inhibited the germination	[224]
*Mentha* × *piperita* L. cv. Mitcham	menthol (35%), mentone (17.48%), menthofuran (11.7%) and 1,8-cineole (5.9%)	EOs	*L. esculentum*, *R. sativus*, *Convolvulus arvensis* L., *P. oleracea* and *E. colonum*	Inhibited germination percentage, root and shoot lengths, and dry weight of the seedlings. Crops were more susceptible than weeds.	[225]
	*trans*-ferulic acid (10.8 mg/g), hesperidin (9.3 mg g^−1^), ellagic acid (6.8 mg g^−1^) and sinapic acid (4.2 mg g^−1^)	Aqueous extract			
*Mentha* × *piperita* L. cv. Mitcham	*trans*-ferulic acid (10.8 mg g^−1^), hesperidin (9.3 mg g^−1^), ellagic acid (6.8 mg g^−1^) and sinapic acid (4.2 mg g^−1^)	Aqueous extract	*R. sativus*	Inhibited germination and growth, total chlorophyll content. Stimulated proline, soluble sugar, phenolic compound content	[148]
*Mentha* × *piperita*	pulegone and menthone	EOs	Cucumber	Root and mitochondrial respiration	[226]
*Thymus algeriensis* Boiss. et Reut.	α-pinene (19.5%), 1,8-cineole (11.6%) and camphor (10.4%)	EOs	*M. sativa* and *T. aestivum*	Inhibited shoot and root growth	[227]
*Thymus fontanesii* Boiss. et Reut.	carvacrol (52.1%), thymol (13.3%), *p*-cymene (12.2%) and γ-terpinene (8.1%)	EOs	*S. arvensis*, *Avena fatua* L., *Sonchus oleraceus* L., *Xanthium strumarium* L. and *C. rotundus*	Inhibited germination percentage	[28]
*Thymus capitatus* Hoff. et Link	carvacrol (63–84%)	EOs	*S. arvensis*	Inhibited germination	[228]
*Thymus capitatus* L.	carvacrol (75.30%)	EOs	Crops: *T. aestivum* and *H. vulgare* Weeds: *L. rigidum* and *P. brachystachys*	Almost no effect on crop’s germination but caused radical length inhibition. However, both germination and radical length of weeds were inhibited	[218]
*Thymus daenensis* Celak.	thymol (20–60.5%) and carvacrol (20.1–63.4%)	EOs	*A. retroflexus*, *Avena fatua*, *Datura stramonium* and *L. sativum*	Inhibited germination	[229]
*Thymus decussatus*	carvacrol (75.91–94.40%)	EOs	*L. sativa*	Inhibited seed germination, shoot, and root growth	[230]
*Thymus capitatus*	carvacrol (68.19%)	EOs	*L. sativa*	Inhibited seed germination	[231]
*Thymus eigii*	thymol (24.77%) and carvacrol (14.0%)	EOs	*L. sativa*, *L. sativum* and *P. oleracea*	Inhibited germination and growth	[232]
*Thymus vulgaris* L.	thymol (35.4%), *p*–cymene (34.7%)	EOs	*P. oleracea*, *Vicia sativa* L.	Inhibited seed germination	[233]
*Thymus capitatus* L., *Thymus vulgaris* L.	*T. capitatus:* thymol (15.17%) and carvacrol (53.16%) *T. vulgaris:* thymol (12.74%) and carvacrol (48.23%)	EOs	*L. sativum*	Inhibited germination parameters (germination percentage, time to get 50% germination, mean germination time, germination index), hypocotyl, and radicle length	[234]
*Hyptis suaveolens* Poit.	14α-hydroxy-13β-abiet-8-en-18-oic acid (suaveolic acid)	Aqueous methanol extract	*L. sativum*, L. *multiflorum* and *E. crus-galli*	Inhibited seedling growth	[32]
*Leucas aspera* (Willd.) Linn.	3:2 mixture of two labdane type diterpenes (rel 5*S*,6*R*,8*R*,9*R*,10*S*,13*S*,15*S*,16*R*)-6-acetoxy-9,13;15,16-diepoxy-15-hydroxy-16-methoxylabdane and (rel 5*S*,6*R*,8*R*,9*R*,10*S*,13*S*,15*R*,16*R*)-6-acetoxy-9,13;15,16-diepoxy-15-hydroxy-16-methoxylabdane (2)	Aqueous methanol extract	*L. sativum* and *E. crus-galli*	Inhibited germination and seedling growth	[30]
*Leonurus sibiricus* L.	caffeic acid	Root exudates	*O. sativa*, *T. aestivum* and *B*. spp.	Inhibited germination and seedling growth	[235]
*Leonurus sibiricus* L.	3′-OH-genkwanin and quercetin	Methanol extract	*L. sativa*	Inhibited germination	[236]
	3′-OH-genkwanin, rutin, and isoquercetrin			Inhibited radicle growth	
*Origanum syriacum*	carvacrol (60.1%), *p*-Cymene (19.7%), γ-Terpinene (13%)	EOs	*T. aestivum* and *Amaranthus*	Inhibited germination	[237]
*Origanum vulgare ssp. vulgare* L.	caryophyllene oxide (34.44%), β-caryophyllene (20.40%) and α-cadinol (7.02%)	EOs	*Z. mays*	DNA alterations	[238]
*Origanum acutidens*	carvacrol (87.0%)	EOs	*A.ret-roflexus*, *C. album*, and *Rumex crispus*	Inhibited seed germination and seedling growth	[86]
*Origanum onites* L.	carvacrol (91.39%)	EOs	Crops: *T. aestivum cv Gün 91*, *H. annuus* cv. Sirena and *C. arietinum* Weeds: *A. retroflexus*, *Rumex crispus* L. and *S. arvensis*	Reduced germination rate of weeds but had no effect on crops	[239]
*Origanum vulgare* L.	carvacrol (34.0%) and γ-terpinene (21.6%), *p*-cymene (9.4%)	EOs	*S. avensis*	Inhibited seed germination and seedling growth	[240]
*Origanum vulgare* L.	methyleugenol (16.5%), myristicin (15.6%), carvacrol (15.0%), thymol (9.8%), and apioline (9.4%)	EOs	*T. aestivum*, *V. radiata* and *R. sativus*	Inhibited seed germination and seedling growth	[241]
*Origanum vulgare* ssp. hirtum	thymol and carvacrol (65.3–84.7%)	EOs	*S. arvensis* L., *P. canariensis*,*L. sativum* L., *and R. sativus* L.	Inhibited seed germination and seedling growth	[242]
*Origanum onites* L.	carvacrol (59.87%), γ-terpinene (17.08%) and β-cymene (8.83%)	EOs	*A. retroflexus* L., *T. aestivum* L. *and L. sativum* L.	Completely inhibited seed germination, and root and shoot growth	[243]
*Rosmarinus officinalis* L.	α-pinene (29.6%), 1,8-cineole (25.6%) and piperitone (14.1%)	Fresh leaves leachate	*Eleusine indica* (L.) Gaertn., *C. dactylon*, *D. sanguinalis*	Inhibited seed germination and seedling growth	[89]
	α-pinene (25.7%), 1,8-cineole (13.2%) and piperitone (20.5%)	Stem			
	α-pinene (33.7%), 1,8-cineole (19.4%) and piperitone (30.4%)	Root			
	α-pinene (44.3%), 1,8-cineole (26.7%) and piperitone (6.5%)	Litter			
	α-pinene, 1,8-cineole, camphor	Aerial parts	*A. retroflexus*, and Lolium *perenne*	Inhibited germination, early growth, and physiological and histological parameters	[39]
	α-pinene (25.8–27.7%), camphor (8.6–9%), camphene (6.5–7.7%) and 1, 8-cineole (9.4–9.6%)		*L. serriola* and *R. sativus*	Inhibited seed germination and growth	[36]
	α-pinene (24.9%), verbenol (8.5%), verbenone (8.5%), 1,8-cineol (8.2%) andisoborneol (8.1%)		*Ruta graveolens* L.	Increased radical elongation at higher concentration (100 µg mL^−1^)	[90]
			*R. sativus*	Inhibited radical elongation at higher concentrations (100 µg mL^−1^)	
			*L. sativa*	Inhibited radical elongation	
			*S. lycopersicum*	Inhibited germination only	
*Satureja hortensis* L.	carvacrol (46.94%) and γ-terpinene (29.14%)	Aerial parts	*L. rigidum* L. and *P. brachystachys* L.	Inhibited the germination and root length of *L. rigidum* and *P. brachystachys*	[218]
*Satureja spp*., *S. khuzestanica*, *S. bachtiarica*, *S. rechingeri* and *S. spicigera*	carvacrol and thymol	EOs	*L. esculentum* and *S. cereale*	*S. khuzestanica* and *S. rechingeri* essential oils showed high inhibitory effect against *L. esculentum* and *S. cereale*, whereas *S. bachtiarica* showed the least. *S. spicigera* and *S. rechingeri* inhibited the germination and growth of *S. cereale*	[107]
*Satureja hortensis* L.	carvacrol (55.6%) and γ-terpinene (31.9%)	EOs nanoemulsion	*A. retroflexus* and *C. album*	Inhibited the germination, shoot-root growth, and chlorophyll content	[244]
*Tectona grandis*	2-oxokovalenic acid and 19-hydroxyferruginol	Aqueous extract dried leaves	*T. aestivum*	Inhibited the elongation of etiolated wheat coleoptiles	[245]
*Orthosiphon stamineus Benth*. (*syn. O. aristatus*, *O. gradiflorus*, *O. spicatus*)	13-epi-Orthosiphol *N*	Shoots	*L. sativum* and *L. sativa*	Inhibited root and hypocotyl growth	[200]
*Calamintha nepeta* L. (Savi)	gallic, vanillic, syringic, *p*-coumaric and ferulic acids from ethylacetate fraction	Methanol extract of leaves and stem	*A. retroflexus* and *E. crus-galli*	Inhibited seed germination and root growth	[29]
	*trans*-caryophyllene, menthol, farnesene and pulegone from *n*-hexane				
	Four terpenoids, camphor, pulegone, *trans*-caryophyllene and farnesene	Methanolic extract and EOs	*Arabidopsis thaliana* (L.) Heynh	Farnesene and trans-caryophyllene had a strong inhibitory effect on root growth, and pulegone at the highest concentrations reduced lateral root formation. The addition, at low concentration, of farnesene to pulegone–camphor–trans-caryophyllene mixture further increased the inhibitory effect on root elongation	[201]
*Calamintha ashei*	Saturated aqueous solutions of menthofuran, (+)-evodone, (-)-calaminthone, (+)-desacetylcalaminthone, 4α,5β−diacetoxymenthofuran, and a mixture of (+)-evodone and (+)-desacetylcalaminthone	Fresh aerial parts	*Schizachyrium scoparium* and *Leptochloa dubia* and *L. sativa*	Inhibited germination and root growth	[246]

**Table 4:** Allelopathic activity of Lamiaceae plant species under pot/greenhouse/field conditions.

Plant Species	Plant Organ	Mode of Application	Type of Experiment	Target Species	Effect	Reference
*Salvia officinalis* L.	Dried leaves biomass	Residue@7.5 t ha^−1^	Green house	*L. esculentum*	Inhibited the shoot length and dry biomass	[262]
		Residue@15 t ha^−1^		*Panicum maximum* Jacq.		
*Ocimum basilicum* L.	Aerial parts	Aqueous extract	*Wire house*	*Amaranthus* and *P. oleraceae*	Reduction in the fresh weight g/pot, and root and stem length	[140]
		Acetone extract (@40.48 kg ha^−1^ which equal 1% extract)	Field	All weeds found in the experimental field	Reduced the fresh weight of different weed species 21 days from spraying.	
	Fresh leaves	Aqueous extract	Pot	*P. minor*, *Anagalis arvensis*	Increasing concentration of up to 25% maximize the inhibitions of both weeds biomass (80%) in two consecutive seasons	[263]
*Lavandula* × *intermedia* cv. Grosso	Coumarin and plant extract	Leaf and stem extract	Cylindrical vials (50% soil: 50% peat moss)	*L. rigidum*	Shoot length and weight were significantly reduced by post-emergence application	[146]
*Mentha* × *piperita* L.	Leaves	Aqueous extracts	Green house	*H. annuus*	Reduced germination and chlorophyll a. Increased electrolyte leakage from seedlings, chlorophyll b, photochemical efficiency of photosystem II	[264]
*Mentha* × *piperita* L.	Mixture of two Mentha varieties	Volatiles	Glasshouse	*B. oleracea* convar. capitata	Stimulated leaf development and dry weight	[265]
*Mentha spicata* L. and *Mentha* × *piperita* L.	Introduction in crop rotation	-	Field experiment	*Z. mays*	Inhibited the plant height, biomass, photosynthetic rate, stomatal conductance, and relative chlorophyllcontent	[266]
*Mentha* sp.	Dry leaves	-	Pot experiment	*B. napus* var. *oleifera*	Stimulated seedling emergence	[267]
*Mentha spicata* L.	Dry above ground biomass	-	Pot experiment	*L. esculentum*	Taller plants with thicker stems, higher chlorophyll content index, and photosynthetic rate and yield.	[268]
*Thymus fontanesii Boiss. et Reut*.	Dried aerial parts	EOs	Greenhouse	*S. arvensis*, *Avena fatua L*., *S. oleraceus*, *X. strumarium* and *C. rotundus*	Wilting, leaf chlorosis, necrotic spots and desiccation, reduced chlorophyll content	[28]
*Thymus* sp.	Thymol	-	Pot experiment	*L. sativa*	Inhibited the shoot fresh and dry weights and photosynthetic rate. Promoted photosystem II, total protein concentration, proline content, antioxidant enzymes (poly-phenol oxidase, ascorbate peroxidase and catalase)	[269]
*Hyptis spicigera*			*Z. mays* was following fallow with *H. spicigera*	*Striga hermonthica*	Reduced *S. hermonthica* incidence and increased *Z. mays* yield	[270]
			Intercropping maize and *H. spicigera*		Negatively affected maize growth, resulting in reduced maize yields	
*Tectona grandis*	Fresh leaves	Leachates	Pot culture	*V. unguiculata*, *M. charantia* and *S. melongena* L.	Inhibited the seed germination and seedling growth	[197]
*Calamintha nepeta* L. (Savi)	Above ground parts	Residue	Pot culture	*L. sativa*, *C. album*, *S. alba*	Inhibited the shoot and root growth	[202]

## Data Availability

Data sharing is not applicable, as no new data were generated or analyzed during this study.
